# Supplementary material for: Role of physical activity in the relationship between recovery from work and insomnia among early childhood education and care professionals: a cross-sectional study
Source: BMJ Open. 2024 Mar 19;14(3):e079746. doi: 10.1136/bmjopen-2023-079746 (PMC10953046; doi:10.1136/bmjopen-2023-079746)
Supplement: Author's manuscript [file bmjopen-2023-079746.draft_revisions.pdf]

BMJ Open is committed to open peer review. As part of this commitment we make the peer review history of every article we publish publicly available.

When an article is published we post the peer reviewers' comments and the authors' responses online. We also post the versions of the paper that were used during peer review. These are the versions that the peer review comments apply to.

The versions of the paper that follow are the versions that were submitted during the peer review process. They are not the versions of record or the final published versions. They should not be cited or distributed as the published version of this manuscript.

BMJ Open is an open access journal and the full, final, typeset and author-corrected version of record of the manuscript is available on our site with no access controls, subscription charges or pay-per-view fees (http://bmjopen.bmj.com).

If you have any questions on BMJ Open's open peer review process please email info.bmjopen@bmj.com

BMJ Open

Role of physical activity in the relationship between need for recovery after work and insomnia among early childhood education and care professionals

Journal:	*BMJ Open*
Manuscript ID	bmjopen-2023-079746
Article Type:	Original research
Date Submitted by the Author:	10-Sep-2023
Complete List of Authors:	Karihtala, Tiina; University of Jyväskylä Faculty of Sports and Health Sciences; Finnish Institute of Occupational Health<br>Puttonen, Sampsa; University of Tampere, Faculty of Social Sciences; Finnish Institute of Occupational Health<br>Valtonen, Anu; Metropolia University of Applied Sciences<br>Kautiainen, Hannu; Kuopio University Hospital, Primary Health Care Unit; Folkhälsan Research Center<br>Hopsu, Leila; Finnish Institute of Occupational Health<br>Heinonen, Ari; University of Jyväskylä, Faculty of Sport and Health Sciences
Keywords:	Occupational Stress, PUBLIC HEALTH, Health Education

SCHOLARONE™
Manuscripts

I, the Submitting Author has the right to grant and does grant on behalf of all authors of the Work (as defined in the below author licence), an exclusive licence and/or a non-exclusive licence for contributions from authors who are: i) UK Crown employees; ii) where BMJ has agreed a CC-BY licence shall apply, and/or iii) in accordance with the terms applicable for US Federal Government officers or employees acting as part of their official duties; on a worldwide, perpetual, irrevocable, royalty-free basis to BMJ Publishing Group Ltd ("BMJ") its licensees and where the relevant Journal is co-owned by BMJ to the co-owners of the Journal, to publish the Work in this journal and any other BMJ products and to exploit all rights, as set out in our licence.

The Submitting Author accepts and understands that any supply made under these terms is made by BMJ to the Submitting Author unless you are acting as an employee on behalf of your employer or a postgraduate student of an affiliated institution which is paying any applicable article publishing charge ("APC") for Open Access articles. Where the Submitting Author wishes to make the Work available on an Open Access basis (and intends to pay the relevant APC), the terms of reuse of such Open Access shall be governed by a Creative Commons licence – details of these licences and which Creative Commons licence will apply to this Work are set out in our licence referred to above.

Other than as permitted in any relevant BMJ Author's Self Archiving Policies, I confirm this Work has not been accepted for publication elsewhere, is not being considered for publication elsewhere and does not duplicate material already published. I confirm all authors consent to publication of this Work and authorise the granting of this licence.

For peer review only

Role of physical activity in the relationship between need for recovery after work and insomnia among early childhood education and care professionals

1. Tiina Karihtala, Faculty of Sport and Health Sciences, University of Jyväskylä, Jyväskylä, Finland and Finnish Institute of Occupational Health, Helsinki, Finland, ORCID: 0000-0002-4353-0955
2. Sampsa Puttonen, Faculty of Social Sciences, University of Tampere, Tampere, Finland and Finnish Institute of Occupational Health, Helsinki, Finland, ORCID: 0000-0002-7796-694
3. Anu M Valtonen, Metropolia University of Applied Sciences, Helsinki, Finland, ORCID: 0000-0002-6214-2085
4. Hannu Kautiainen, Primary Health Care Unit, Kuopio University Hospital, Kuopio, Finland and Folkhälsan Research Center, Helsinki, Finland, ORCID: 0000-0003-0786-0858
5. Leila Hopsu, Finnish Institute of Occupational Health, Helsinki, Finland
6. Ari Heinonen, Faculty of Sport and Health Sciences, University of Jyväskylä, Jyväskylä, Finland, ORCID: 0000-0002-3681-9953

For peer review only

Abstract

Objectives This study aimed to investigate the association between need for recovery after work and insomnia and the role of objectively measured leisure-time physical activity and occupational physical activity in this association.

Design Cross-sectional.

Participants Female early childhood education and care professionals (N = 224) in Finland with a mean age of 44 (SD 11) years.

Methods Need for recovery after work was measured with the Need for Recovery scale and insomnia with the Jenkins Sleep Scale. Physical activity was measured with an accelerometer during waking hours for seven consecutive days and analysed separately to represent leisure time physical activity and occupational physical activity (min/day).

Results Both Jenkins Sleep Scale and occupational physical activity significantly predicted need for recovery ($\beta = 0.29$; 95% CI: 0.17 to 0.42 and $\beta = 0.14$; 95% CI: 0.01 to 0.27, respectively). A moderate relationship was observed between the need for recovery and Jenkins Sleep Scale ($r = 0.32$, 95% Cl: 0.19 to 0.44). After categorizing participants into four groups based on median splits of occupational and leisure-time physical activity, relationships between the need for recovery and Jenkins Sleep Scale were moderate in the high occupational physical activity and leisure time physical activity group ($r = 0.38$, 95% Cl 0.14 to 0.61), as well as in the high occupational physical activity and low leisure time physical activity group ($r = 0.40$, 95% Cl 0.18 to 0.63).

Conclusion Both insomnia and physical activity at work were related to the need for recovery after work. Insomnia and need for recovery are suggested to relate especially when physical activity is high during working hours. To enhance recovery from work and maintain good work ability, the importance of good sleep is emphasized. Attention should also be paid to the amount and intensity of physical activity during free time.

For peer review only - http://bmjopen.bmj.com/site/about/guidelines.xhtml

Keywords
need for recovery after work, insomnia, occupational physical activity, leisure time physical activity, accelerometer

Strengths and limitations of this study

- In this study, an accelerometer was used as an objective tool to measure physical activity.
- Participants represent well early childhood education and care workers in Finland.
- The study was cross-sectional, and longitudinal studies are needed to examine the temporal relationships.
- The findings of this study may not be directly applicable to other occupations or to men.

Word count 3225

Declarations

Ethics approval and consent to participate: Ethical approval for the study was granted by the Helsinki University Central Hospital Coordinating Ethics Committee (HUCH/1883/2016). Written informed consent was obtained from all participants prior to enrolment.

Consent for publication: Not applicable

Availability of data: The data are available from the first author upon reasonable request and with permission of Finnish Institute of Occupational Health, Helsinki, Finland.

Patient and public involvement: Patients and/or the public were not involved in the design, or conduct, or reporting, or dissemination plans of this research.

Competing interests: None declared.

Funding: This work was supported by the Academy of Finland (287195), University of Jyväskylä, Faculty of Sport and Health Sciences, Otto A. Malm Foundation, Helsinki, Finland.

Author' contributions:

All authors whose names appear on the submission

1. Made a significant contribution to the work reported. That could be in the conception, study design, execution, acquisition of data, analysis and interpretation, or in all these areas.

2. Have drafted or written, substantially revised or critically reviewed the article.

3. Have agreed on the journal to which the article will be submitted.

4. Reviewed and agreed on all versions of the article before submission, during revision, the final version accepted for publication, and any significant changes introduced at the proofing stage.

For peer review only

5. Agree to take responsibility and be accountable for the contents of the article. Share responsibility to resolve any questions raised about the accuracy or integrity of the published work.

Acknowledgements: Not applicable

For peer review only

For peer review only - http://bmjopen.bmj.com/site/about/guidelines.xhtml

Background

Changes in working life, such as increased work intensity and difficulty of separating work from leisure time, challenge relaxation and recovery after working hours. Recovery from work is essential for an individual's work ability and health (1,2), and more attention should be drawn to the activities during non-work time to unwind from work-induced stress (3). Successful recovery ensures sufficient energy for the next day's work activities and for meaningful free time or off-work responsibilities. On the other hand, lack of recovery can decrease work ability and increase the risk of early exit from work life (1,4). The experience of insufficient recuperation from work and the need to take a break from ongoing activity is referred to as 'need for recovery after work' (5,6).

Demerouti et al. (7) have suggested that the success of recuperating from workload is affected by the demands and resources both at work and at home and also by sleep and leisure-time activities. Sufficient and high-quality sleep enables brain and body to recover from physiological and psychological workload (8,9). In contrast, insufficient sleep and insomnia have diverse health-deteriorating effects (10–14). In addition, poor sleep quality decreases work ability (15) and is linked to increased sickness absences (16). Yet, the association between insomnia and need for recovery after work has received little attention. Preliminary evidence suggests that insomnia is associated with higher need for recovery (17,18).

We (19) and Stevens et al. (4) showed that a high level of accelerometer-measured occupational physical activity (OPA) is associated with increased need for recovery after work among early childhood education and care (ECEC) professionals and blue-collar workers. Contradictory results have been reported for office employees and health care workers in studies with self-reported physical activity (20,21). On the other hand, leisure-time physical activity (LTPA) has been suggested to enhance recovery from work by supporting detachment from work (22,23). However, we did not observe this among ECEC professionals (19). To reduce discrepancies between studies and increase the reliability of the results, methodologies for physical activity measures should be unified; recommendations to implement objective measures have been presented (24,25).

Systematic reviews have found positive associations between LTPA and sleep quality, sleep onset latency and sleep duration (26–28). However, the association between LTPA and insomnia was not detected in two other recent systematic reviews (29,30). Regarding OPA, studies have found that a high level of physical activity during working hours is related to insomnia (31–35) and that LTPA

For peer review only - http://bmjopen.bmj.com/site/about/guidelines.xhtml

may protect against insomnia even when high OPA exists (33). Since the effects of OPA and LTPA on insomnia seem to diverge, it is important to examine physical activity in these two domains separately.

In supporting employee well-being, it is important to understand how on-work and off-work activities, including sleep, can promote recovery. So far, little research exists on the association between need for recovery after work and insomnia, and, to our knowledge, research among ECEC professionals in this regard is non-existing. Also, the role of physical activity during work or leisure time in this context is not yet fully understanded. The aims of this study were, first, to investigate the association between need for recovery after work and insomnia, and, secondly, to study the roles of accelerometer-measured leisure-time and occupational physical activity in this association in female ECEC professionals. Our hypothesis was that insomnia is associated with need for recovery after work, that LTPA would enhance recovery and that OPA would deteriorate it.

Methods

Design and participants

This study is part of the DagisWork study (workplace healthcare interventions to promote the work ability of kindergarten personnel, NCT03854877). It was conducted during 2017–2019 in two southern Finland cities. In total, there are 218 ECEC centres in the area, and 78 volunteered to participate. Finally, a random sample of 23 centres with 386 participants were included in the study. Exclusion criteria were set as retirement or termination of employment within next six months or pregnancy. Hence, 269 participants signed the informed consent form and joined the study. Men were excluded because of their limited number (n = 3). Participants were not involved in designing, conducting, reporting, or dissemination plans of this study. At the workplace, two health care professionals performed baseline measurements (weight, height, body mass index) and instructed participants on the use of a waist-worn accelerometer for the following week. Concurrent filling in a diary as well as an electronic questionnaire was guided. The 224 participants with complete data for need for recovery after work, insomnia and physical activity at baseline formed the final analytical sample. Ethical approval for the study was granted by the Helsinki University Central Hospital coordinating ethics committee (HUCH/1883/2016).

For peer review only - http://bmjopen.bmj.com/site/about/guidelines.xhtml

Recovery

Recovery was assessed with the Need for Recovery (NFR) scale (6,36), which examines perceived need for recovery after work. Items in the scale represent reduced performance, feeling of overload, irritability, social withdrawal and lack of energy for new tasks, with statements such as the following: “I find it difficult to relax at the end of a working day”, “I find it difficult to concentrate in my free time after work” or “When I go home from work, I need to be left in peace for a while”. The final score of 0–100 was comprised of 11 “yes” or “no” answers in 11 dichotomous questions and was calculated as a percentage of positive answers. The higher the percentage score was, the higher was need for recovery after work. The scale was implemented in the electronic questionnaire, and at least 8 answers out of 11 was required in order to be included in the analysis.

Insomnia

Insomnia symptoms were assessed using the Jenkins Sleep Scale (JSS) (37), a widely used tool to assess insomnia symptoms in the working-age population (38,39) with good psychometric properties in the Finnish version (40). Participants were asked about difficulties falling asleep, waking up several times per night, difficulties staying asleep and non-restorative sleep (i.e., feeling tired and worn-out waking up after the usual amount of sleep) during the previous month. The sum score of 4–24 was comprised from the responses given on the scale, from 1 to 6 (1 = not at all, 2 = 1–3 nights/month, 3 = 1 night/week, 4 = 2–4 nights/week, 5 = 5–6 nights/week, 6 = every night).

Physical activity

Physical activity was measured with the waist-worn accelerometer (ActiGraph GT9X Link, ActiGraph, USA) for seven consecutive days and nights. The meter was only removed for showering or other water-related activities. At the same time, participants recorded working hours and sleep times in a diary. Awake time was separated into occupational and leisure time. Participants with data for at least four days and 10 hours/day were eligible for the final analysis. ActiLife software (6.13.3) was used for data analysis, and 30 Hz frequency and 60-second epochs

For peer review only - http://bmjopen.bmj.com/site/about/guidelines.xhtml

were used. Non-wear time criteria from Choi et al. (41) were applied, and a minimum length of 30 minutes and a drop time of 2 minutes for sedentary time were used. Cut points for different thresholds for physical activity intensity levels were used as follows: sedentary 0–99 counts per minute (cpm), light 100–1951 cpm, moderate 1952–5724 cpm, vigorous 5725–9498 cpm, and very vigorous over 9499 cpm (42). Average minutes/day were calculated for each intensity level. For analysis, physical activity was defined as any level of physical activity (light, moderate, vigorous or very vigorous). Mean physical activity minutes at different levels were added up and, with the help of diaries, calculated separately for leisure time to represent LTPA and for occupational time to represent OPA.

Other variables

The electronic questionnaire was used to register *demographics* and *other characteristics* of the participants, such as marital status, smoking (yes/no), alcohol consumption (units/week), educational level (university degree / vocational degree) and ECEC work experience (years).

Work ability was reported with a single question: "How good is your current work ability compared to your lifetime best?" and was rated on a scale from 0 to 10 (0 = completely unable to work; 10 = work ability at its best) (43).

Mental health symptoms were measured with the General Health Questionnaire (GHQ-12) (44). The 12 questions with response choices being "much more than usual", "rather more than usual", "no more than usual" and "not at all", were scored from 0 to 3, respectively. A sum score was calculated (0–36).

Perceived health was examined with one question: "In general, would you say your health is?" Answers were given on a scale from 1 = poor to 5 = excellent, and values 4 and 5 were classified as "good health" (45).

Stress level was measured with the Perceived Stress Scale (PSS-10) including 10 questions such as "In the last month, how often have you felt nervous and stressed?" rated on a 5-point scale (0–4) and added up as a sum score of 0–40 (46).

For peer review only - http://bmjopen.bmj.com/site/about/guidelines.xhtml

For peer review only

Musculoskeletal, cardiovascular, respiratory, or mental disorders were registered with open response questions and dichotomized to yes or no, with the classification "yes" requiring both diagnosis by a doctor and symptoms being present at the moment or occurring often or repeatedly.

Pain intensity during the last month was registered on 1-to-6 scale, from no pain to very severe pain, and *pain interference* on a 1-to-5 scale, from no pain to daily pain. Both scales were obtained from the Short Form 36 Health Survey Questionnaire pain (45) and were converted to a score of 0 to 100.

Statistical analysis

Data are expressed as mean and standard deviation (SD) or frequencies with percentages. The relationship between FMI and LMI was modelled using linear regression analysis. Results were analysed using factorial (two between-subjects factors: LTPA and OPA) analysis of variance (ANOVA) and logistic models. Models included main effects of LTPA and OPA and their interaction. Multivariate linear regression analysis was used to identify the relationship between LTPA, OPA, JSS and age as continuous variables and the need for recovery after work with standardized regression coefficient Beta (β). The Beta value is a measure of how strongly the predictor variable influences the criterion variable. The Beta is measured in units of SD. Cohen's standard for Beta values above 0.10, 0.30 and 0.50 represents small, moderate and large relationships, respectively. Unadjusted and adjusted (partial) correlations were calculated by the Pearson method, using Sidak adjusted confidence interval. Stata 17.0 (StataCorp LP; College Station, Texas, USA) statistical package was used for the analysis.

For peer review only

Results

The mean age of the participants was 44 (SD 11) years, and 65% of them had been working more than 10 years in an ECEC centre. They represented the typical employee groups in Finnish ECEC centres with managers (7%), special education teachers (6%), teachers (36%), child carers (42%), and assistants (9%).

The scatter plot in Figure 1 shows the relationship between LTPA and OPA among the participants. Table 1-displays participant characteristics in the four physical activity groups, based on the median values of LTPA and OPA. No interactions between LTPA and OPA were detected in any of the

For peer review only - http://bmjopen.bmj.com/site/about/guidelines.xhtml

variables. The results showed that high LTPA was related with a longer career history at an ECEC centre and low OPA to a higher level of education (Table 1).

Figure 1 here

Table 1. Demographic and clinical characteristics of the participants according to low or high leisure time physical activity (LTPA, min/day) and occupational physical activity (OPA, min/day).

	high LTPA		low LTPA		p-value		
	low OPA	high OPA	low OPA	high OPA	Main effect		Interaction
	n = 53	n = 59	n = 58	n = 54	LTPA	OPA	
Age, mean (SD) years	44(9)	45(11)	44(11)	43(12)	0.57	0.95	0.60
In partnership, n (%)	36(71)	40(75)	39(70)	38(73)	0.36	0.83	0.90
Smoker, n (%)	7(14)	11(21)	8(14)	9(17)	0.81	0.33	0.72
Alcohol use[a], mean (SD)	2.0(2.5)	2.7(3.6)	2.1(3.2)	1.6(2.1)	0.22	0.72	0.15
Highly educated, n (%)	29(57)	21(40)	31(56)	14(27)	0.30	<0.001	0.33
Career history in an ECEC centre (years)					0.009	0.51	0.37
<1	2(4)	2(4)	0(0)	2(4)			
1–3	2(4)	5(9)	7(13)	8(15)			
4–10	10(20)	6(11)	15(27)	16(31)			
>10	37(73)	40(75)	34(61)	26(50)			
BMI, mean (SD)	27.3(7.8)	27.0(5.2)	26.7(6.1)	27.5(5.6)	0.99	0.72	0.54
Work ability, mean (SD)	8.0(1.3)	8.2(1.1)	8.1(1.3)	8.4(0.9)	0.49	0.19	0.78
GHQ-12, mean (SD)	12.4(5.6)	12.5(5.0)	12.0(5.0)	11.2(4.0)	0.21	0.63	0.48
Good health, self-rated, n (%)	33(65)	35(66)	37(66)	29(56)	0.51	0.51	0.39
Perceived Stress Scale, mean (SD)	16.7(6.5)	16.3(6.6)	16.9(6.2)	14.8(6.4)	0.45	0.14	0.34
Disorders, n (%)							
Musculoskeletal	14(27)	17(32)	14(25)	8(16)	0.11	0.58	0.22
Cardiovascular	9(18)	8(15)	9(16)	7(14)	0.77	0.63	0.99

For peer review only - http://bmjopen.bmj.com/site/about/guidelines.xhtml

Respiratory	6(12)	6(11)	9(16)	6(12)	0.63	0.63	0.70
Mental	1(2)	5(9)	7(13)	3(6)	0.27	0.53	0.06
Pain (SF36), mean (SD)							
Intensity	33(23)	32(22)	33(22)	33(24)	0.93	0.96	0.92
Interference	25(21)	24(26)	25(25)	23(25)	0.78	0.67	0.88
OPA min/day, mean (SD)	190(31)	267(32)	184(37)	269(25)	..	..	..
LTPA min/day, mean (SD)	305(57)	295(41)	196(42)	212(23)	..	..	..
Need for recovery after work, mean (SD)	35.8(28.3)	41.5(28.9)	37.4(23.6)	34.9(24.4)	0.50	0.66	0.27
Jenkins Sleep Scale, mean (SD)	6.8(4.3)	6.3(4.1)	6.8(3.9)	6.7(5.0)	0.70	0.59	0.65
difficulty falling asleep	*0.7(1.0)*	*0.9(1.0)*	*1.3(1.2)*	*1.0(1.2)*			
waking up several times per night	*2.4(1.6)*	*2.5(1.5)*	*2.4(1.6)*	*2.3(1.7)*			
difficulty staying asleep	*1.7(1.6)*	*1.8(1.5)*	*1.4(1.4)*	*1.7(1.6)*			
non-restorative sleep	*2.3(1.4)*	*1.8(1.3)*	*2.0(1.3)*	*2.2(1.4)*			

[a]units per week (1 unit = 12 grams of pure alcohol), Highly educated = university level degree, ECEC = early childhood education and care, BMI = body mass index, GHQ-12 = General Health Questionnaire, good health = 3–5 (on a scale of 0–5), SF36 = Short Form 36 Health Survey Questionnaire, OPA = occupational physical activity, LTPA = leisure time physical activity.

Need for recovery and insomnia

Participants' average NFR score was 37.5% (SD 26.3), and 30% of participants were classified as having high need for recovery (>54.5%). The mean JSS value was 6.7 (SD 4.3), and 10% of participants suffered from insomnia disorder, defined as difficulty falling asleep, waking up several times per night, difficulty staying asleep, or non-restorative sleep occurring five or more nights per week. The mean time for LTPA was 252 min/day (SD 66) and for OPA 228 min/day (SD 51).

A moderate relationship was observed between NFR and JSS sum score (r = 0.32, 95% Cl: 0.19 to 0.44). Table 2 shows the low to moderate relationships between NFR and individual JSS symptoms including waking up several times per night, difficulty staying asleep, and non-restorative sleep.

For peer review only - http://bmjopen.bmj.com/site/about/guidelines.xhtml

Table 2. Correlations between the need for recovery after work (NFR) and individual insomnia symptoms measured by the Jenkins Sleep Scale (JSS).

	r	95% CI*
Difficulty to fall asleep	0.17	0.04 to 0.30
Waking up several times per night	0.14	0.00 to 0.27
Difficulty to stay asleep	0.26	0.13 to 0.38
Non-restorative sleep	0.38	0.26 to 0.49

* Confidence intervals adjusted following the Sidak method.

Age-adjusted correlations between NFR and JSS for the four physical activity groups are presented in Figure 2. Relationships between NFR and JSS were moderate in the high OPA and LTPA group (r = 0.38, 95% Cl 0.14 to 0.61), as well as in the high OPA and low LTPA group (r = 0.40, 95% Cl 0.18 to 0.63). Correlations were small in the low OPA and LTPA group (r = 0.20, 95% Cl -0.02 to 0.43), as well as in the low OPA and high LTPA group (r = 0.29, 95% Cl 0.02 to 0.51). Hence, NFR was related to insomnia, especially if physical activity during the working day was high.

Figure 2 here

Multivariate relationships between NFR and LTPA, OPA, age and JSS are presented in Figure 3. Both JSS and OPA significantly predicted NFR, but the standardized regression coefficients were small (β = 0.29; CI: 0.17 to 0.42 and β = 0.14; 95% CI: 0.01 to 0.27, respectively).

Figure 3 here

Discussion

In this study, we asked whether the amount of physical activity during working hours or leisure time influence the association between recovery and insomnia in female ECEC professionals. The results supported our hypothesis regarding the impairing role of occupational physical activity in the recovery process. Need for recovery after work was related to both insomnia and physical activity during working hours. Further, need for recovery after work and insomnia associated

For peer review only - http://bmjopen.bmj.com/site/about/guidelines.xhtml

particularly when workers' occupational activity was high. However, the findings did not support our hypothesis about the recovery-enhancing role of physical activity during leisure time.

Average NFR among the female ECEC professionals was 38%. This is slightly higher than in airline workers (males 20% and females 28%), but substantially lower than in emergency department staff (82%) (47,48). We found that 30% of ECEC professionals had high NFR (>54.5%), which is in line with earlier studies with nurses and kindergarten teachers (30%) and industry and health care workers (21%) (21,49). The prevalence of insomnia has been reported to vary internationally between 11.3% and 41.5% (50–52). In our study, 46% of participants reported occasional insomnia symptoms during the previous month (any insomnia symptom 2–4 nights per week) and 10% suffered from insomnia disorder (any insomnia symptom ≥5 nights per week); the readings are very similar to those of a previous Finnish study (45% and 10%, respectively) (53).

ECEC professionals spent 58% of their waking time sedentary, 37% in light, 5% in moderate and, not at all (0.002%) in vigorous physical activity. A recent large cohort study reported similar accelerometer-derived physical activity level proportions: 55%, 39%, 5% and 0%, respectively (54). In high-income Western countries, the prevalence of insufficient physical activity is estimated to be 42% (55), meaning that just over half of the population meet the recommendation of physical activity for adults (U.S. Department of Health and Human Services, 2018). In our study, participants spent 47 minutes per day in moderate to vigorous physical activities, which is almost three times more than that of child care staff in the United States (17 minutes) (56).

The results of this study suggest that insomnia is related to increased need for recovery after work. Our results are in line with previous studies on this topic (17,18). The association was detected with several insomnia symptoms (waking up several times per night, difficulties staying asleep, and non-restorative sleep) reflecting diverse deficiencies with sleep health that might challenge recovery. Even one night of sleep deprivation has been shown to increase need for recovery for the following two days (57). This emphasizes the importance of a regular sleep rhythm and sufficient sleep for recovery after work. However, surprisingly few studies have explored the association between the need for recovery after work and insomnia. More studies focusing on sleep health in promoting recovery from work are warranted. Also, longitudinal studies are needed to examine cause-and-effect relationships in more detail.

For peer review only - http://bmjopen.bmj.com/site/about/guidelines.xhtml

We found that, in addition to insomnia, physical activity during working hours was associated with recovery need. Insomnia and need for recovery were related especially when the amount of physical activity during the working day was high. This result suggests that even the observed low to moderate ECEC centre workload is relevant in this context. Only a few previous studies have also linked high physical workload during work to sleep difficulties (31,35,58). Further, physically demanding work and sleep difficulties have been found to associate with poorer physical function (59,60). We did not measure participants' physical function; therefore, it remains unclear whether even a light workload, especially if long-lasting, may be too wearing and increase the need for recovery after work when one's physical condition is weak. To our knowledge, the importance of physical activity during the working day in this context has not been previously reported. However, our result can only be considered preliminary. In this cross-sectional study, we investigated only women, and they all worked in the same occupational setting (i.e. an ECEC centre). The research should be repeated with men, as well as with different professions, including those with more physically demanding work tasks.

The results suggest that the level of LTPA is not relevant to the relationship between insomnia and need for recovery after work. Earlier studies have linked high LTPA levels to better sleep (27,61) while the appropriate duration and intensity of physical activity are still debated (27). It has also been suggested that a high level of LTPA may protect from insomnia even when activity during the working day is high (33). However, this was not confirmed by our results. In some studies, high leisure-time physical activity has been linked to a lower need for recovery (20,62). Theoretically, we can assume that LTPA improves physical fitness, which increases stress tolerance and thereby reduces the need for recovery after work. On the other hand, LTPA may facilitate relaxation, promote psychological detachment from work and enhance experience of having autonomy and control over leisure-time choices and activities (3). However, our results did not support these assumptions. First, this may be due to the fact that accelerometer-derived LTPA inadequately captured participants' leisure-time activities performed in stationary positions, such as strength training. Second, an accelerometer may not be able to capture activities unwinding from work, such as relaxation exercises or Pilates-type training, for example. Third, the level and intensity of LTPA among ECEC workers may have been too low to improve physical fitness and thereby increase load tolerance at work. Also, what forms of exercise would best enhance recovery from work remains unknown. In future, research should consider different domains of LTPA suitable at an individual level to enhance unwinding from work. For that, diary or similar methods would be needed parallel to technological measures. Also, longitudinal studies are needed to explore which domains of

For peer review only - http://bmjopen.bmj.com/site/about/guidelines.xhtml

physical activity best promote recovery and to find individually suitable dose-response LTPA relationships.

Conclusions

In conclusion, both insomnia and the amount of physical activity during a working day are related to the need for recovery after work. Insomnia and need for recovery are related especially if physical activity is high during a working day. This means that in order to maintain good work ability and prevent a high need for recovery after work, sufficient and high-quality sleep should be ensured, especially with ECEC professionals with a high level of physical activity during working days. Moreover, attention should be paid to sufficient level and intensity of leisure-time activities to improve physical fitness and thus load and stress tolerance at work.

References

1. Stynen D, Jansen NWH, Slangen JJM, de Grip A, Kant IJ. Need for recovery and different types of early labour force exit: a prospective cohort study among older workers. Int Arch Occup Environ Health. 2019;92(5):683–97.

2. van Hooff ML, Geurts SA, Taris TW, Kompier MA, Dikkers JS, Houtman IL, et al. Disentangling the causal relationships between work-home interference and employee health. Scand J Work Environ Health. 2005;31(1):15–29.

3. Sonnentag S, Cheng BH, Parker SL. Recovery from Work: Advancing the Field Toward the Future. Annu Rev Organ Psychol Organ Behav. 2022;9(1):33–60.

4. Stevens ML, Crowley P, Rasmussen CL, Hallman DM, Mortensen OS, Nygård CH, et al. Accelerometer-Measured Physical Activity at Work and Need for Recovery: A Compositional Analysis of Cross-sectional Data. Ann Work Expo Health. 2020;64(2):138–51.

5. Sonnentag S, Zijlstra FRH. Job characteristics and off-job activities as predictors of need for recovery, well-being, and fatigue. J Appl Psychol. 2006;91(2):330–50.

6. van Veldhoven M. Measurement quality and validity of the "need for recovery scale." Occup Environ Med. 2003;60:3i–9.

For peer review only - http://bmjopen.bmj.com/site/about/guidelines.xhtml

7. Demerouti E, Bakker AB, Geurts SAE, Taris TW. Daily recovery from work-related effort during non-work time. In: Sonnentag S, Perrewé PL, Ganster DC, editors. Research in Occupational Stress and Well-being. Emerald Group Publishing Limited; 2009:85–123.

8. Garrick A, Mak AS, Cathcart S, Winwood PC, Bakker AB, Lushington K. Non-Work Time Activities Predicting Teachers' Work-Related Fatigue and Engagement: An Effort-Recovery Approach. Aust Psychol. 2018;53(3):243–52.

9. Xie L, Kang H, Xu Q, Chen MJ, Liao Y, Thiyagarajan M, et al. Sleep Drives Metabolite Clearance from the Adult Brain. Science. 2013;342(6156):373–7.

10. Cabeza de Baca T, Chayama KL, Redline S, Slopen N, Matsushita F, Prather AA, et al. Sleep debt: the impact of weekday sleep deprivation on cardiovascular health in older women. Sleep. 2019;42(10):1–8.

11. Cappuccio FP, Cooper D, D'Elia L, Strazzullo P, Miller MA. Sleep duration predicts cardiovascular outcomes: a systematic review and meta-analysis of prospective studies. Eur Heart J. 2011;32(12):1484–92.

12. Hertenstein E, Feige B, Gmeiner T, Kienzler C, Spiegelhalder K, Johann A, et al. Insomnia as a predictor of mental disorders: A systematic review and meta-analysis. Sleep Med Rev. 2019;43:96–105.

13. Jike M, Itani O, Watanabe N, Buysse DJ, Kaneita Y. Long sleep duration and health outcomes: A systematic review, meta-analysis and meta-regression. Sleep Med Rev. 2018;39:25–36.

14. Wu TT, Zou YL, Xu KD, Jiang XR, Zhou MM, Zhang SB, et al. Insomnia and multiple health outcomes: umbrella review of meta-analyses of prospective cohort studies. Public Health. 2023;215:66–74.

15. Bergman E, Löyttyniemi E, Myllyntausta S, Rautava P, Korhonen PE. Factors associated with quality of life and work ability among Finnish municipal employees: a cross-sectional study. BMJ Open. 2020;10(9):e035544.

16. Lallukka T, Kaikkonen R, Härkänen T, Kronholm E, Partonen T, Rahkonen O, et al. Sleep and Sickness Absence: A Nationally Representative Register-Based Follow-Up Study. Sleep. 2014;37(9):1413–25.

17. Rook JW, Zijlstra FRH. The contribution of various types of activities to recovery. Eur J Work Organ Psychol. 2006;15(2):218–40.

18. Silva-Costa A, Griep RH, Fischer FM, Rotenberg L. Need for recovery from work and sleep-related complaints among nursing professionals. Work. 2012;41:3726–31.

19. Karihtala T, Valtonen AM, Kautiainen H, Hopsu L, Halonen J, Heinonen A, et al. Relationship between occupational and leisure-time physical activity and the need for recovery after work. Arch Public Health. 2023;81(1):17.

20. Coffeng JK, van Sluijs EM, Hendriksen IJM, van Mechelen W, Boot CRL. Physical Activity and Relaxation During and After Work are Independently Associated With the Need for Recovery. J Phys Act Health. 2015;12(1):109–15.

For peer review only

For peer review only - http://bmjopen.bmj.com/site/about/guidelines.xhtml

21. Gommans FG, Jansen NWH, Mackey MG, Stynen D, de Grip A, Kant Ij. The Impact of Physical Work Demands on Need for Recovery, Employment Status, Retirement Intentions, and Ability to Extend Working Careers: A Longitudinal Study Among Older Workers. J Occup Environ Med. 2016;58(4):e140–51.

22. van Hooff MLM, Benthem de Grave RM, Geurts SAE. No pain, no gain? Recovery and strenuousness of physical activity. J Occup Health Psychol. 2019;24(5):499–511.

23. Korpela K, Kinnunen U. How Is Leisure Time Interacting with Nature Related to the Need for Recovery from Work Demands? Testing Multiple Mediators. Leis Sci. 2010;33(1):1–14.

24. Steene-Johannessen J, Anderssen SA, Van Der Ploeg HP, Hendriksen IJM, Donnelly AE, Brage S, et al. Are Self-report Measures Able to Define Individuals as Physically Active or Inactive? Med Sci Sports Exerc. 2016;48(2):235–44.

25. Van Poel E, Ketels M, Clays E. The association between occupational physical activity, psychosocial factors and perceived work ability among nurses. J Nurs Manag. 2020;28(7):1696–703.

26. Fank F, Pereira F da S, dos Santos L, de Mello MT, Mazo GZ. Effects of Exercise on Sleep in Older Adults: An Overview of Systematic Reviews and Meta-Analyses. J Aging Phys Act. 2022;1–17.

27. Wang F, Boros S. The effect of physical activity on sleep quality: a systematic review. Eur J Physiother. 2021;23(1):11–8.

28. Wendt A, Flores TR, Silva ICM, Wehrmeister FC. Association of physical activity with sleep health: A systematic review. Rev Bras Atividade Física Saúde. 2018;23:1–26.

29. Memon AR, Gupta CC, Crowther ME, Ferguson SA, Tuckwell GA, Vincent GE. Sleep and physical activity in university students: A systematic review and meta-analysis. Sleep Med Rev. 2021;58:101482.

30. Atoui S, Chevance G, Romain AJ, Kingsbury C, Lachance JP, Bernard P. Daily associations between sleep and physical activity: A systematic review and meta-analysis. Sleep Med Rev. 2021;57:101426.

31. Beak M, Choi WJ, Lee W, Ham S. Associations of Abnormal Sleep Duration with Occupational and Leisure-time Physical Activity in the Working Population: A Nation-wide Population-based Study. Saf Health Work. 2021;12(3):311–6.

32. Lim M, Jeong KS, Oh SS, Koh SB, Chang SJ, Ahn YS. Effects of Occupational and Leisure-Time Physical Activities on Insomnia in Korean Firefighters. Int J Environ Res Public Health Electron Resour. 2020;17(15).

33. Skarpsno ES, Mork PJ, Nilsen TIL, Jørgensen MB, Holtermann A. Objectively measured occupational and leisure-time physical activity: cross-sectional associations with sleep problems. Scand J Work Environ Health. 2018;44(2):202–211.

34. Skarpsno ES, Mork PJ, Nilsen TIL, Jørgensen MB, Holtermann A. The joint association of musculoskeletal pain and domains of physical activity with sleep problems: cross-sectional data from the DPhacto study, Denmark. Int Arch Occup Environ Health. 2019;92(4):491–9.

For peer review only

35. Wennman H, Kronholm E, Partonen T, Tolvanen A, Peltonen M, Vasankari T, et al. Physical activity and sleep profiles in Finnish men and women. BMC Public Health. 2014;14(1):82.

36. de Croon EM. Psychometric properties of the Need for Recovery after work scale: test-retest reliability and sensitivity to detect change. Occup Environ Med. 2006;63(3):202–6.

37. Jenkins CD, Stanton BA, Niemcryk SJ, Rose RM. A scale for the estimation of sleep problems in clinical research. J Clin Epidemiol. 1988;41(4):313–21.

38. Lallukka T, Dregan A, Armstrong D. Comparison of a Sleep Item From the General Health Questionnaire-12 With the Jenkins Sleep Questionnaire as Measures of Sleep Disturbance. J Epidemiol. 2011;21(6):474–80.

39. Tibubos AN, Zenger M, Schmalbach B, Beutel ME, Brähler E. Measurement invariance, validation and normative data of the Jenkins Sleep Scale-4 (JSS-4) in the German general population across the life span. J Psychosom Res. 2020;130:109933.

40. Juhola J, Arokoski JPA, Ervasti J, Kivimäki M, Vahtera J, Myllyntausta S, et al. Internal consistency and factor structure of Jenkins Sleep Scale: cross-sectional cohort study among 80 000 adults. BMJ Open. 2021;11(1):e043276.

41. Choi L, Liu Z, Matthews CE, Buchowski MS. Validation of Accelerometer Wear and Nonwear Time Classification Algorithm. Med Sci Sports Exerc. 2011;43(2):357–64.

42. Freedson PS, Melanson E, Sirard J. Calibration of the Computer Science and Applications, Inc. accelerometer. Med Sci Sports Exerc. 1998;30(5):777.

43. Ilmarinen J. Work ability—a comprehensive concept for occupational health research and prevention. Scand J Work Environ Health. 2009;35(1):1–5.

44. Goldberg DP, Gater R, Sartorius N, Ustun TB, Piccinelli M, Gureje O, et al. The validity of two versions of the GHQ in the WHO study of mental illness in general health care. Psychol Med. 1997;27(1):191–7.

45. Ware JE, Sherbourne CD. Erratum to The MOS 36-item short form health survey (SF-36): I. Conceptual framework. PharmacoEconomics. 1992;2(2):98–98.

46. Cohen S, Kamarck T, Mermelstein R. A Global Measure of Perceived Stress. J Health Soc Behav. 1983;24(4):385.

47. Graham B, Cottey L, Smith JE, Mills M, Latour JM. Measuring 'Need for Recovery' as an indicator of staff well-being in the emergency department: a survey study. Emerg Med J. 2020;37(9):555–61.

48. Hemiö K, Lindström J, Peltonen M, Härmä M, Viitasalo K, Puttonen S. High need for recovery from work and sleep problems are associated with workers' unhealthy dietary habits. Public Health Nutr. 2021;24(8):2185–94.

49. Qi X, Liu Y, Zhang J, Ji S, Sluiter JK, Zhou R, et al. Relationship between work strain, need for recovery after work and cumulative cortisol among kindergarten teachers. Int Arch Occup Environ Health. 2015;88(8):1053–9.

50. Aernout E, Benradia I, Hazo JB, Sy A, Askevis-Leherpeux F, Sebbane D, et al. International study of the prevalence and factors associated with insomnia in the general population. Sleep Med. 2021;82:186–92.

51. Appleton SL, Reynolds AC, Gill TK, Melaku YA, Adams RJ. Insomnia Prevalence Varies with Symptom Criteria Used with Implications for Epidemiological Studies: Role of Anthropometrics, Sleep Habit, and Comorbidities. Nat Sci Sleep. 2022;14:775–90.

52. van de Straat V, Bracke P. How well does Europe sleep? A cross-national study of sleep problems in European older adults. Int J Public Health. 2015;60(6):643–50.

53. Kronholm E, Partonen T, Härmä M, Hublin C, Lallukka T, Peltonen M, et al. Prevalence of insomnia-related symptoms continues to increase in the Finnish working-age population. J Sleep Res. 2016;25(4):454–7.

54. Ekblom-Bak E, Börjesson M, Bergman F, Bergström G, Dahlin-Almevall A, Drake I, et al. Accelerometer derived physical activity patterns in 27.890 middle-aged adults: The SCAPIS cohort study. Scand J Med Sci Sports. 2022;32(5):866–80.

55. Guthold R, Stevens GA, Riley LM, Bull FC. Worldwide trends in insufficient physical activity from 2001 to 2016: a pooled analysis of 358 population-based surveys with 1·9 million participants. Lancet Glob Health. 2018;6(10):e1077–86.

56. Ward DS, Vaughn AE, Hales D, Viera AJ, Gizlice Z, Bateman LA, et al. Workplace health and safety intervention for child care staff: Rationale, design, and baseline results from the CARE cluster randomized control trial. Contemp Clin Trials. 2018;68:116–26.

57. Laharnar N, Fatek J, Zemann M, Glos M, Lederer K, Suvorov AV, et al. A sleep intervention study comparing effects of sleep restriction and fragmentation on sleep and vigilance and the need for recovery. Physiol Behav. 2020;215:112794.

58. Åkerstedt T, Fredlund P, Gillberg M, Jansson B. Work load and work hours in relation to disturbed sleep and fatigue in a large representative sample. J Psychosom Res. 2002;53(1):585–8.

59. Aikomus L, Lehtinen-Jacks S, Nordquist H, Pietiläinen O, Mänty M, Kouvonen A, et al. Associations between physically demanding work and physical functioning among municipal employees under 40 years of age. Sosiaalilääketieteellinen aikakauslehti – Journal of Social Medicine 2021:58;445–456.

60. Miranda H, Viikari-Juntura E, Punnett L, Riihimäki H. Occupational loading, health behavior and sleep disturbance as predictors of low-back pain. Scand J Work Environ Health. 2008;34(6):411–9.

61. Bláfoss R, Sundstrup E, Jakobsen MD, Bay H, Garde AH, Andersen LL. Are Insomnia Type Sleep Problems Associated With a Less Physically Active Lifestyle? A Cross-Sectional Study Among 7,700 Adults From the General Working Population. Front Public Health. 2019;7:117.

62. Wilson I, McDermott H, Munir F. The role of working hours, work environment and physical leisure activity on the need for recovery following a day's work among UK white-water raft guides: A within-subjects multilevel approach. Psychol Sport Exerc. 2016;23:123–31.

For peer review only - http://bmjopen.bmj.com/site/about/guidelines.xhtml

Legends for figures:

Figure 1. Daily minutes of physical activity categorized into four groups based on median splits of leisure time physical activity (LTPA min/day) and occupational physical activity (OPA min/day): A) high LTPA + low OPA, B) high LTPA + high OPA, C) low LTPA + low OPA and D) low LTPA + high OPA. Medians for LTPA (246.2 min/day) and for OPA (231.0 min/day) are illustrated as dashed lines.

Figure 2. Age-adjusted correlations with 95% CIs between Need for Recovery and Jenkins Sleep Scale for the physical activity groups based on low or high leisure-time physical activity (LTPA, min/day) and occupational physical activity (OPA, min/day): high LTPA + low OPA, high LTPA + high OPA, low LTPA + low OPA or low LTPA + high OPA.

Figure 3. Multivariate relationships between Need for Recovery and occupational physical activity (OPA) level, leisure-time physical activity (LTPA) level, age, and Jenkins Sleep Scale (JSS) sum score. Cohen's standards for beta values above 0.10, 0.30, and 0.50 represent small, moderate, and large effect sizes, respectively.

For peer review only

For peer review only - http://bmjopen.bmj.com/site/about/guidelines.xhtml

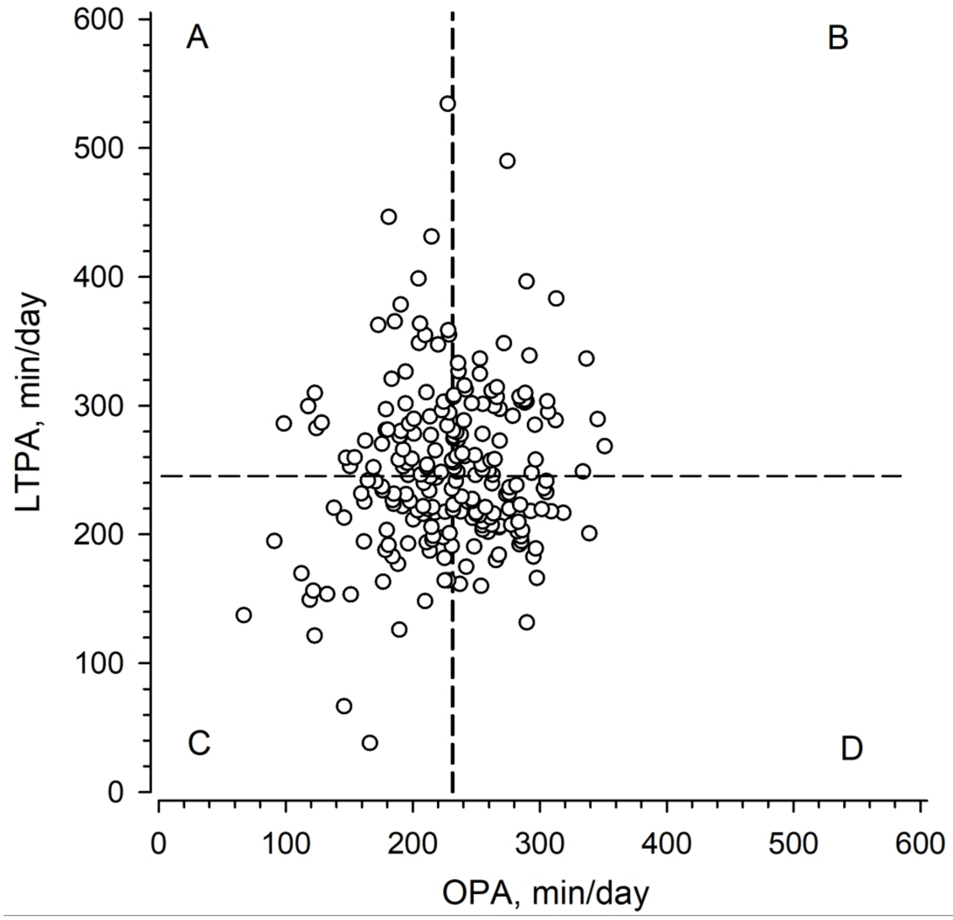

Figure 1. Daily minutes of physical activity categorized into four groups based on median splits of leisure time physical activity (LTPA min/day) and occupational physical activity (OPA min/day): A) high LTPA + low OPA, B) high LTPA + high OPA, C) low LTPA + low OPA and D) low LTPA + high OPA. Medians for LTPA (246.2 min/day) and for OPA (231.0 min/day) are illustrated as dashed lines.

226x208mm (236 x 236 DPI)

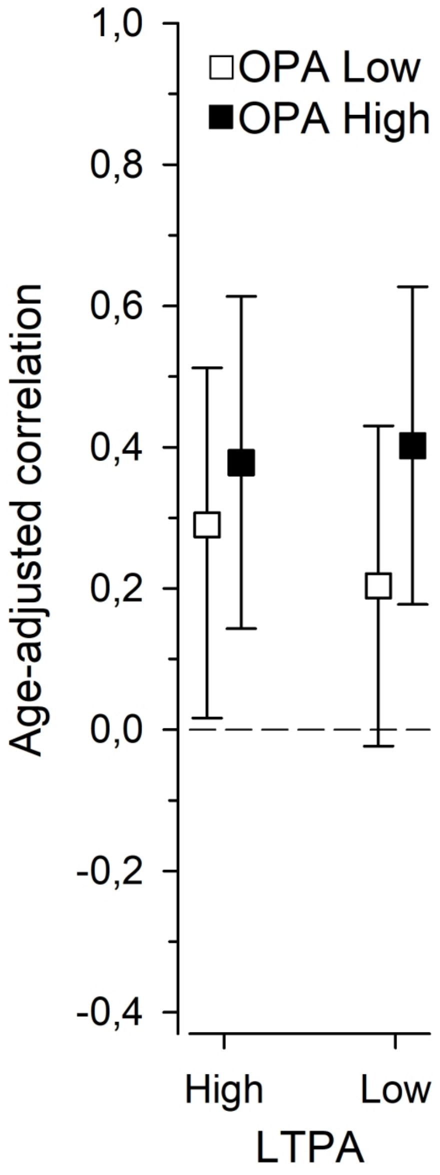

Figure 2. Age-adjusted correlations with 95% CIs between Need for Recovery and Jenkins Sleep Scale for the physical activity groups based on low or high leisure-time physical activity (LTPA, min/day) and occupational physical activity (OPA, min/day): high LTPA + low OPA, high LTPA + high OPA, low LTPA + low OPA or low LTPA + high OPA.

105x271mm (236 x 236 DPI)

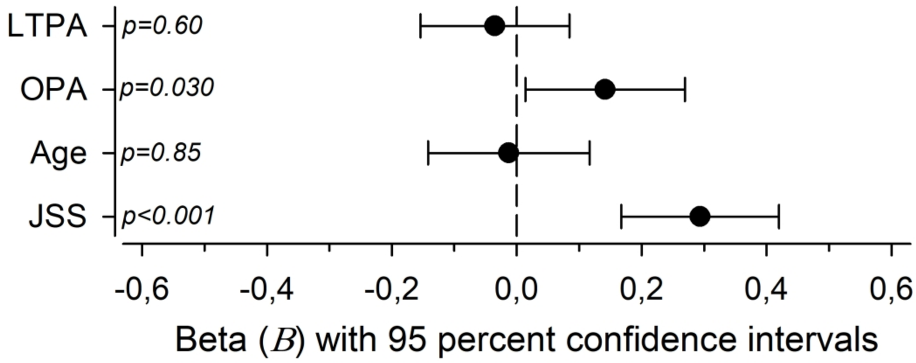

Figure 3. Multivariate relationships between Need for Recovery and occupational physical activity (OPA) level, leisure-time physical activity (LTPA) level, age, and Jenkins Sleep Scale (JSS) sum score. Cohen's standards for beta values above 0.10, 0.30, and 0.50 represent small, moderate, and large effect sizes, respectively.

278x133mm (236 x 236 DPI)

STROBE checklist

Section/Topic	Item #	Recommendation	Reported on page #
**Title and abstract**	1	(*a*) Indicate the study's design with a commonly used term in the title or the abstract	1
		(*b*) Provide in the abstract an informative and balanced summary of what was done and what was found	1
**Introduction**			
Background/rationale	2	Explain the scientific background and rationale for the investigation being reported	3 and 4
Objectives	3	State specific objectives, including any prespecified hypotheses	4
**Methods**			
Study design	4	Present key elements of study design early in the paper	4
Setting	5	Describe the setting, locations, and relevant dates, including periods of recruitment, exposure, follow-up, and data collection	4
Participants	6	(*a*) Give the eligibility criteria, and the sources and methods of selection of participants	4
Variables	7	Clearly define all outcomes, exposures, predictors, potential confounders, and effect modifiers. Give diagnostic criteria, if applicable	4 and 5
Data sources/ measurement	8*	For each variable of interest, give sources of data and details of methods of assessment (measurement). Describe comparability of assessment methods if there is more than one group	4,5 and 6
Bias	9	Describe any efforts to address potential sources of bias	4
Study size	10	Explain how the study size was arrived at	4
Quantitative variables	11	Explain how quantitative variables were handled in the analyses. If applicable, describe which groupings were chosen and why	4,5 and 6
Statistical methods	12	(*a*) Describe all statistical methods, including those used to control for confounding	6
		(*b*) Describe any methods used to examine subgroups and interactions	6
		(*c*) Explain how missing data were addressed	4
		(*d*) If applicable, describe analytical methods taking account of sampling strategy	4
		(*e*) Describe any sensitivity analyses	NA

For peer review only

**Results**			
Participants	13*	(a) Report numbers of individuals at each stage of study—eg numbers potentially eligible, examined for eligibility, confirmed eligible, included in the study, completing follow-up, and analysed	4 and supplement
		(b) Give reasons for non-participation at each stage	4
		(c) Consider use of a flow diagram	supplement
Descriptive data	14	(a) Give characteristics of study participants (eg demographic, clinical, social) and information on exposures and potential confounders	8
		(b) Indicate number of participants with missing data for each variable of interest	4
Outcome data	15	Report numbers of outcome events or summary measures	NA
Main results	16	(*a*) Give unadjusted estimates and, if applicable, confounder-adjusted estimates and their precision (eg, 95% confidence interval). Make clear which confounders were adjusted for and why they were included	9 and 10
		(*b*) Report category boundaries when continuous variables were categorized	8
		(*c*) If relevant, consider translating estimates of relative risk into absolute risk for a meaningful time period	NA
Other analyses	17	Report other analyses done—eg analyses of subgroups and interactions, and sensitivity analyses	NA
**Discussion**			
Key results	18	Summarise key results with reference to study objectives	11
Limitations	19	Discuss limitations of the study, taking into account sources of potential bias or imprecision. Discuss both direction and magnitude of any potential bias	11 and 12
Interpretation	20	Give a cautious overall interpretation of results considering objectives, limitations, multiplicity of analyses, results from similar studies, and other relevant evidence	11 and 12
Generalisability	21	Discuss the generalisability (external validity) of the study results	12
**Other information**			
Funding	22	Give the source of funding and the role of the funders for the present study and, if applicable, for the original study on which the present article is based	2

For peer review only

BMJ Open

Role of physical activity in the relationship between recovery from work and insomnia among early childhood education and care professionals: a cross-sectional study

Journal:	*BMJ Open*
Manuscript ID	bmjopen-2023-079746.R1
Article Type:	Original research
Date Submitted by the Author:	01-Feb-2024
Complete List of Authors:	Karihtala, Tiina; University of Jyväskylä Faculty of Sports and Health Sciences; Finnish Institute of Occupational Health<br>Puttonen, Sampsa; University of Tampere, Faculty of Social Sciences; Finnish Institute of Occupational Health<br>Valtonen, Anu; Metropolia University of Applied Sciences<br>Kautiainen, Hannu; Kuopio University Hospital, Primary Health Care Unit; Folkhälsan Research Center<br>Hopsu, Leila; Finnish Institute of Occupational Health<br>Heinonen, Ari; University of Jyväskylä, Faculty of Sport and Health Sciences
**Primary Subject Heading**:	Public health
Secondary Subject Heading:	Public health
Keywords:	Occupational Stress, PUBLIC HEALTH, Health Education

SCHOLARONE™
Manuscripts

I, the Submitting Author has the right to grant and does grant on behalf of all authors of the Work (as defined in the below author licence), an exclusive licence and/or a non-exclusive licence for contributions from authors who are: i) UK Crown employees; ii) where BMJ has agreed a CC-BY licence shall apply, and/or iii) in accordance with the terms applicable for US Federal Government officers or employees acting as part of their official duties; on a worldwide, perpetual, irrevocable, royalty-free basis to BMJ Publishing Group Ltd ("BMJ") its licensees and where the relevant Journal is co-owned by BMJ to the co-owners of the Journal, to publish the Work in this journal and any other BMJ products and to exploit all rights, as set out in our licence.

The Submitting Author accepts and understands that any supply made under these terms is made by BMJ to the Submitting Author unless you are acting as an employee on behalf of your employer or a postgraduate student of an affiliated institution which is paying any applicable article publishing charge ("APC") for Open Access articles. Where the Submitting Author wishes to make the Work available on an Open Access basis (and intends to pay the relevant APC), the terms of reuse of such Open Access shall be governed by a Creative Commons licence – details of these licences and which Creative Commons licence will apply to this Work are set out in our licence referred to above.

Other than as permitted in any relevant BMJ Author's Self Archiving Policies, I confirm this Work has not been accepted for publication elsewhere, is not being considered for publication elsewhere and does not duplicate material already published. I confirm all authors consent to publication of this Work and authorise the granting of this licence.

For peer review only

Role of physical activity in the relationship between recovery from work and insomnia among early childhood education and care professionals: a cross-sectional study

1. Tiina Karihtala, Faculty of Sport and Health Sciences, University of Jyväskylä, Jyväskylä, Finland and Finnish Institute of Occupational Health, Helsinki, Finland, ORCID: 0000-0002-4353-0955
2. Sampsa Puttonen, Faculty of Social Sciences, University of Tampere, Tampere, Finland and Finnish Institute of Occupational Health, Helsinki, Finland, ORCID: 0000-0002-7796-694
3. Anu M Valtonen, Metropolia University of Applied Sciences, Helsinki, Finland, ORCID: 0000-0002-6214-2085
4. Hannu Kautiainen, Primary Health Care Unit, Kuopio University Hospital, Kuopio, Finland and Folkhälsan Research Center, Helsinki, Finland, ORCID: 0000-0003-0786-0858
5. Leila Hopsu, Finnish Institute of Occupational Health, Helsinki, Finland
6. Ari Heinonen, Faculty of Sport and Health Sciences, University of Jyväskylä, Jyväskylä, Finland, ORCID**:** 0000-0002-3681-9953

Corresponding author: Tiina Karihtala, tiina.karihtala@metropolia.fi

For peer review only

Abstract

Objectives This study aimed to investigate the association between recovery from work and insomnia and the role of objectively measured leisure-time physical activity and occupational physical activity in this association.

Design Cross-sectional.

Setting and participants Study with female early childhood education and care professionals (N = 224) in Finland was conducted between April 2017 and September 2018.

Methods Recovery from work was measured with the Need for Recovery scale and insomnia with the Jenkins Sleep Scale. Physical activity was measured with an accelerometer for seven days and analysed to represent leisure time physical activity and occupational physical activity (min/day).

Results Both Jenkins Sleep Scale and occupational physical activity significantly predicted Need for Recovery ($\beta = 0.29$; 95% CI: 0.17 to 0.42 and $\beta = 0.14$; 95% CI: 0.01 to 0.27, respectively). A low relationship was observed between the Need for Recovery and Jenkins Sleep Scale ($r = 0.32$, 95% Cl: 0.19 to 0.44). After categorizing participants into four groups based on median splits of occupational and leisure-time physical activity, relationships between the Need for Recovery and Jenkins Sleep Scale were low to moderate in the high occupational physical activity and leisure time physical activity group ($r = 0.38$, 95% Cl 0.14 to 0.61), and in the high occupational physical activity and low leisure time physical activity group ($r = 0.40$, 95% Cl 0.18 to 0.63).

Conclusion Both insomnia and physical activity at work seem to be relevant in recovery from work. To enhance recovery, especially those involved in high physical activity at work, should seek methods to improve recovery, by incorporating activities that promote recuperation both during their workday and in their leisure time. Further research on the relevance of physical activity in recovery with longitudinal setting are warranted.

For peer review only - http://bmjopen.bmj.com/site/about/guidelines.xhtml

Keywords
recovery from work, insomnia, occupational physical activity, leisure time physical activity, accelerometer

Strengths and limitations of this study

- In this study, an accelerometer was used as an objective tool to measure physical activity.
- Participants represent well early childhood education and care workers in Finland.
- The study was cross-sectional, and longitudinal studies are needed to examine the temporal relationships.
- The findings of this study may not be directly applicable to other occupations or to men.

Word count 3968

For peer review only

Declarations

Ethics approval and consent to participate: Ethical approval for the study was granted by the Helsinki University Central Hospital Coordinating Ethics Committee (HUCH/1883/2016). Written informed consent was obtained from all participants prior to enrolment.

Consent for publication: Not applicable

Availability of data: The data are available from the first author upon reasonable request and with permission of Finnish Institute of Occupational Health, Helsinki, Finland.

Competing interests: None declared.

Funding: This work was supported by the Academy of Finland (287195), University of Jyväskylä, Faculty of Sport and Health Sciences (Grant no: NA), Otto A. Malm Foundation, Helsinki, Finland (Grant no: NA).

Author' contributions: TK, SP, AV, HK, AH were responsible for constructing an idea, research question and hypothesis for the study. TK and LH collected the data. Initial data processing was done by TK and HK was responsible for final statistics and figures. TK was responsible for initial manuscript and SP, AV, HK, LH and AH have read, critically revised and approved the final version of this manuscript.

Acknowledgements: Not applicable

For peer review only - http://bmjopen.bmj.com/site/about/guidelines.xhtml

Background

Changes in working life, such as increased work intensity and difficulty of separating work from leisure time, challenge relaxation and recovery after working hours. Recovery from work is essential for an individual's work ability and health (1,2), and more attention should be drawn to the activities during non-work time to unwind from work-induced stress (3). Successful recovery ensures sufficient energy for the next day's work activities and for meaningful free time or off-work responsibilities. On the other hand, lack of recovery can decrease work ability and increase the risk of early exit from work life (1,4). The experience of insufficient recuperation from work and the need to take a break from ongoing activity is referred to as 'need for recovery from work' (5,6).

Demerouti et al. (7) have suggested that the success of recuperating from workload is affected by the demands and resources both at work and at home and also by sleep and leisure-time activities. Sufficient and high-quality sleep enables brain and body to recover from physiological and psychological workload (8,9). In contrast, insufficient sleep and insomnia have diverse health-deteriorating effects (10–14). In addition, poor sleep quality decreases work ability (15) and is linked to increased sickness absences (16). Yet, the association between insomnia and recovery from work has received little attention. Preliminary evidence suggests that insomnia is associated with higher need for recovery from work (17,18).

We (19) and Stevens et al. (4) showed that a high level of accelerometer-measured occupational physical activity (OPA) is associated with increased need for recovery from work among early childhood education and care (ECEC) professionals and blue-collar workers. Contradictory results have been reported for office employees and health care workers in studies with self-reported physical activity (20,21). On the other hand, leisure-time physical activity (LTPA) has been suggested to enhance recovery from work by supporting detachment from work (22,23). However, we did not observe this among ECEC professionals (19). To reduce discrepancies between studies and increase the reliability of the results, methodologies for physical activity measures should be unified; recommendations to implement objective measures have been presented (24,25).

Systematic reviews have found positive associations between LTPA and sleep quality, sleep onset latency and sleep duration (26–28). However, the association between LTPA and insomnia was not detected in two other recent systematic reviews (29,30). Regarding OPA, studies have found that a high level of physical activity during working hours is related to insomnia (31–35) and that LTPA

For peer review only - http://bmjopen.bmj.com/site/about/guidelines.xhtml

may protect against insomnia even when high OPA exists (33). Since the effects of OPA and LTPA on insomnia seem to diverge, it is important to examine physical activity in these two domains separately.

In supporting employee well-being, it is important to understand how on-work and off-work activities, including sleep, can promote recovery. So far, little research exists on the association between recovery from work and insomnia, and, to our knowledge, research among ECEC professionals in this regard is non-existing. Also, the role of physical activity during work or leisure time in this context is not yet fully understood. The aims of this study were, firstly, to investigate the association between recovery from work and insomnia, and, secondly, to study the roles of accelerometer-measured leisure-time and occupational physical activity in this association in female ECEC professionals. Our hypothesis was that insomnia is associated with need for recovery from work, that LTPA would enhance recovery and that OPA would deteriorate it.

Methods

Design and participants

This cross-sectional study is a secondary analysis of the DagisWork study, a randomized controlled trial (workplace healthcare interventions to promote the work ability of kindergarten personnel, NCT03854877). It was conducted during 2017–2018 in 23 ECEC centres in two southern Finland cities. Study profile is presented in Figure 1. Centres with less than seven workers were excluded due to the estimated excessive need of time and resources for baseline measurements. Exclusion criteria for a single participant were retirement or termination of employment within next six months, or pregnancy. Men were excluded because of their limited number (n = 3). Finally, random sample of 23 centers with altogether eligible 386 employees were selected to the study. Hence, 117 individuals did not give consent and 269 participants continued with baseline measurements (response rate 70%). Participants were not involved in designing, conducting, reporting, or dissemination plans of this study. At the workplace, two health care professionals performed baseline measurements (weight, height, body mass index) and instructed participants on the use of a waist-worn accelerometer for the following week. Concurrent filling in a diary as well as an electronic questionnaire was guided. The 224 participants with complete data for recovery from work, insomnia and physical activity at baseline formed the final analytical sample. Ethical approval for the study

For peer review only - http://bmjopen.bmj.com/site/about/guidelines.xhtml

was granted by the Helsinki University Central Hospital coordinating ethics committee (HUCH/1883/2016).

Figure1 here

Recovery from work

Recovery from work was assessed with the Need for Recovery (NFR) scale, which is known to be valid and reliable in assessing need to recuperate from work (ICCs 0.68 to 0.80, effect size 0.40) (6,36). Items in the scale represent reduced performance, feeling of overload, irritability, social withdrawal and lack of energy for new tasks, with statements such as the following: "I find it difficult to relax at the end of a working day", "I find it difficult to concentrate in my free time after work" or "When I go home from work, I need to be left in peace for a while". The final score of 0–100 was comprised of 11 "yes" or "no" answers in 11 dichotomous questions and was calculated as a percentage of positive answers. The higher the percentage score was, the higher was need for recovery from work. The scale was implemented in the electronic questionnaire, and at least 8 answers out of 11 was required in order to be included in the analysis.

Insomnia

Insomnia symptoms were assessed using the Jenkins Sleep Scale (JSS) (37), a widely used tool to assess insomnia symptoms in the working-age population (38,39) with good psychometric properties in the Finnish version (40). Participants were asked about difficulties falling asleep, waking up several times per night, difficulties staying asleep and non-restorative sleep (i.e., feeling tired and worn-out waking up after the usual amount of sleep) during the previous month. The sum score of 4–24 was comprised from the responses given on the scale, from 1 to 6 (1 = not at all, 2 = 1–3 nights/month, 3 = 1 night/week, 4 = 2–4 nights/week, 5 = 5–6 nights/week, 6 = every night).

Physical activity

For peer review only - http://bmjopen.bmj.com/site/about/guidelines.xhtml

Physical activity was measured with the waist-worn accelerometer (ActiGraph GT9X Link, ActiGraph, USA) for seven consecutive days and nights. During the nights the meter was worn in the wrist. The meter was only removed for showering or other water-related activities. At the same time, participants recorded working hours and sleep times in a diary. Awake time was separated into occupational and leisure time. Participants with data for at least four days and 10 hours/day were eligible for the final analysis. ActiLife software (6.13.3) was used for data analysis, and 30 Hz frequency and 60-second epochs were used. Non-wear time criteria from Choi et al. (41) were applied, and a minimum length of 30 minutes and a drop time of 2 minutes for sedentary time were used. Cut points for different thresholds for physical activity intensity levels were used as follows: sedentary 0–99 counts per minute (cpm), light 100–1951 cpm, moderate 1952–5724 cpm, vigorous 5725–9498 cpm, and very vigorous over 9499 cpm (42). Counts per minute refer to the number of times that an accelerometer detects movement (change in acceleration). Sedentary time was excluded from data and average PA minutes/day were calculated for each PA intensity levels. For analysis, physical activity was defined as any level of physical activity (light, moderate, vigorous or very vigorous) and mean physical activity minutes/day at all PA intensity levels were added up. With the help of diaries, PA minutes were calculated separately for leisure time to represent LTPA and for occupational time to represent OPA. Later in this report high time (= high min/day) spent on LTPA or OPA will be referred as high LTPA or OPA.

Other variables

The electronic questionnaire was used to register *demographics* and *other characteristics* of the participants, such as marital status, smoking (yes/no), alcohol consumption (units/week), educational level (university degree / vocational degree) and ECEC work experience (years).

Work ability was reported with a single question: "How good is your current work ability compared to your lifetime best?" and was rated on a scale from 0 to 10 (0 = completely unable to work; 10 = work ability at its best) (43).

Mental health symptoms were measured with the General Health Questionnaire (GHQ-12) (44). The 12 questions with response choices being "much more than usual", "rather more than usual", "no more than usual" and "not at all", were scored from 0 to 3, respectively. A sum score was calculated (0–36).

For peer review only

For peer review only - http://bmjopen.bmj.com/site/about/guidelines.xhtml

Perceived health was examined with one question: “In general, would you say your health is?” Answers were given on a scale from 1 = poor to 5 = excellent, and values 4 and 5 were classified as “good health” (45).

Stress level was measured with the Perceived Stress Scale (PSS-10) including 10 questions such as “In the last month, how often have you felt nervous and stressed?” rated on a 5-point scale (0–4) and added up as a sum score of 0–40 (46).

Musculoskeletal, cardiovascular, respiratory, or mental disorders were registered with open response questions and dichotomized to yes or no, with the classification “yes” requiring both diagnosis by a doctor and symptoms being present at the moment or occurring often or repeatedly.

Pain intensity during the last month was registered on 1-to-6 scale, from no pain to very severe pain, and *pain interference* on a 1-to-5 scale, from no pain to daily pain. Both scales were obtained from the Short Form 36 Health Survey Questionnaire pain (45) and were converted to a score of 0 to 100.

Statistical analysis

Data are expressed as mean and standard deviation (SD) or frequencies with percentages. The relationship between NFR and JSS was modelled using linear regression analysis. Results were analysed using factorial (two between-subjects factors: LTPA and OPA) analysis of variance (ANOVA) and logistic models. Models included main effects of LTPA and OPA and their interaction. Multivariate linear regression analysis was used to identify the relationship between LTPA, OPA, JSS and age as continuous variables and the NFR with standardized regression coefficient Beta (β). The Beta value is a measure of how strongly the predictor variable influences the criterion variable. The Beta is measured in units of SD. Cohen’s standard for Beta values above 0.10, 0.30 and 0.50 represents small, moderate and large relationships, respectively. Unadjusted and adjusted (partial) correlations were calculated by the Pearson method, using Sidak adjusted confidence interval. Stata 17.0 (StataCorp LP; College Station, Texas, USA) statistical package was used for the analysis.

Patient and public involvement

None.

For peer review only

For peer review only - http://bmjopen.bmj.com/site/about/guidelines.xhtml

Results

The mean age of all participants was 44 (SD 11) years, and 65% of them had been working more than 10 years in an ECEC centre. They represented the typical employee groups in Finnish ECEC centres with managers (7%), special education teachers (6%), teachers (36%), child carers (42%), and assistants (9%). Participants' average NFR score was 37.5% (SD 26.3), and 30% of participants were classified as having high need for recovery from work (>54.5%). The mean JSS score was 6.7 (SD 4.3), and 10% of participants suffered from insomnia disorder (any insomnia symptom ≥5 nights /week). The mean time for LTPA was 252 min/day (SD 66) and for OPA 228 min/day (SD 51).

Association between recovery from work and insomnia in all participants

A low relationship was observed between NFR and JSS sum score (r = 0.32, 95% Cl: 0.19 to 0.44). Table 1 shows the relationships between NFR and individual JSS symptoms including waking up several times per night, difficulty staying asleep, and non-restorative sleep.

Table 1. Correlations between the Need for Recovery (NFR) and individual insomnia symptoms measured by the Jenkins Sleep Scale (JSS).

	r	95% CI*
Difficulty to fall asleep	0.17	0.04 to 0.30
Waking up several times per night	0.14	0.00 to 0.27
Difficulty to stay asleep	0.26	0.13 to 0.38
Non-restorative sleep	0.38	0.26 to 0.49

* Confidence intervals adjusted following the Sidak method.

Background characteristics

Participants were divided into four physical activity groups according to median values of LTPA (min/day) and OPA (min/day) (Figure 2.).

Figure 2 here

Participant characteristics are presented in these groups in Table 2. showing how physical activity is reflected in the characteristics of different PA groups. No interactions between LTPA and OPA were

For peer review only - http://bmjopen.bmj.com/site/about/guidelines.xhtml

detected in any of the variables. The results showed that high LTPA was related with a longer career history at an ECEC centre and low OPA to a higher level of education.

Table 2. Demographic and clinical characteristics of the participants according to low or high leisure time physical activity (LTPA, min/day) and occupational physical activity (OPA, min/day).

	high LTPA		low LTPA		p-value		
	low OPA	high OPA	low OPA	high OPA	Main effect		Interaction
	n = 53	n = 59	n = 58	n = 54	LTPA	OPA	
Age, mean (SD) years	44(9)	45(11)	44(11)	43(12)	0.57	0.95	0.60
In partnership, n (%)	36(71)	40(75)	39(70)	38(73)	0.36	0.83	0.90
Smoker, n (%)	7(14)	11(21)	8(14)	9(17)	0.81	0.33	0.72
Alcohol use[a], mean (SD)	2.0(2.5)	2.7(3.6)	2.1(3.2)	1.6(2.1)	0.22	0.72	0.15
Highly educated, n (%)	29(57)	21(40)	31(56)	14(27)	0.30	<0.001	0.33
Career history in an ECEC centre (years)					0.009	0.51	0.37
<1	2(4)	2(4)	0(0)	2(4)			
1–3	2(4)	5(9)	7(13)	8(15)			
4–10	10(20)	6(11)	15(27)	16(31)			
>10	37(73)	40(75)	34(61)	26(50)			
BMI, mean (SD)	27.3(7.8)	27.0(5.2)	26.7(6.1)	27.5(5.6)	0.99	0.72	0.54
Work ability, mean (SD)	8.0(1.3)	8.2(1.1)	8.1(1.3)	8.4(0.9)	0.49	0.19	0.78
GHQ-12, mean (SD)	12.4(5.6)	12.5(5.0)	12.0(5.0)	11.2(4.0)	0.21	0.63	0.48
Good health, self-rated, n (%)	33(65)	35(66)	37(66)	29(56)	0.51	0.51	0.39
Perceived Stress Scale, mean (SD)	16.7(6.5)	16.3(6.6)	16.9(6.2)	14.8(6.4)	0.45	0.14	0.34
Disorders, n (%)							
Musculoskeletal	14(27)	17(32)	14(25)	8(16)	0.11	0.58	0.22
Cardiovascular	9(18)	8(15)	9(16)	7(14)	0.77	0.63	0.99
Respiratory	6(12)	6(11)	9(16)	6(12)	0.63	0.63	0.70
Mental	1(2)	5(9)	7(13)	3(6)	0.27	0.53	0.06
Pain (SF36), mean (SD)							
Intensity	33(23)	32(22)	33(22)	33(24)	0.93	0.96	0.92
Interference	25(21)	24(26)	25(25)	23(25)	0.78	0.67	0.88
OPA min/day, mean (SD)	190(31)	267(32)	184(37)	269(25)	..	..	..
LTPA min/day, mean (SD)	305(57)	295(41)	196(42)	212(23)	..	..	..
Need for Recovery mean (SD)	35.8(28.3)	41.5(28.9)	37.4(23.6)	34.9(24.4)	0.50	0.66	0.27
Jenkins Sleep Scale, mean (SD)	6.8(4.3)	6.3(4.1)	6.8(3.9)	6.7(5.0)	0.70	0.59	0.65
difficulty falling asleep	*0.7(1.0)*	*0.9(1.0)*	*1.3(1.2)*	*1.0(1.2)*			

For peer review only

waking up several times per night	*2.4(1.6)*	*2.5(1.5)*	*2.4(1.6)*	*2.3(1.7)*			
difficulty staying asleep	*1.7(1.6)*	*1.8(1.5)*	*1.4(1.4)*	*1.7(1.6)*			
non-restorative sleep	*2.3(1.4)*	*1.8(1.3)*	*2.0(1.3)*	*2.2(1.4)*			

[a]units per week (1 unit = 12 grams of pure alcohol), Highly educated = university level degree, ECEC = early childhood education and care, BMI = body mass index, GHQ-12 = General Health Questionnaire, good health = 3–5 (on a scale of 0–5), SF36 = Short Form 36 Health Survey Questionnaire, OPA = occupational physical activity, LTPA = leisure time physical activity.

Relationship between recovery from work and insomnia in four physical activity groups

Age-adjusted correlations between NFR and JSS for the high and low LTPA and OPA groups are presented in Figure 3. Relationships between NFR and JSS were low to moderate in the high OPA and LTPA group (r = 0.38, 95% Cl 0.14 to 0.61), and the high OPA and low LTPA group (r = 0.40, 95% Cl 0.18 to 0.63). Correlations were low in the low OPA and LTPA group (r = 0.20, 95% Cl -0.02 to 0.43), and in the low OPA and high LTPA group (r = 0.29, 95% Cl 0.02 to 0.51). Hence, results indicate that NFR is related to insomnia, especially if physical activity during the working day is high.

Figure 3 here

Relationship between recovery from work, physical activity and insomnia

Multivariate relationships between NFR and LTPA, OPA, age and JSS are presented in Figure 4. Both JSS and OPA significantly predicted NFR, but the standardized regression coefficients were small (β = 0.29; CI: 0.17 to 0.42 and β = 0.14; 95% CI: 0.01 to 0.27, respectively).

Figure 4 here

Discussion

We investigated the relationship between the recovery from work and insomnia, exploring whether physical activity has role on this relation in female ECEC professionals. Findings suggest that there is a relation between recovery from work and both insomnia and physical activity during working hours. The results supported our hypothesis regarding the detrimental relation of occupational physical activity on recovery, as recovery from work and insomnia were particularly associated with

For peer review only

For peer review only - http://bmjopen.bmj.com/site/about/guidelines.xhtml

the groups engaged in higher physical activity during work, compared to those with lower work-related activity. However, contrary to our hypothesis, the findings did not support the idea of recovery enhancing role of leisure time physical activity.

Average NFR among the female ECEC professionals was 38%. This is slightly higher than in airline workers (males 20% and females 28%), but substantially lower than in emergency department staff (82%) (47,48). We found that 30% of ECEC professionals had high NFR (>54.5%), similar to earlier studies with nurses and kindergarten teachers (30%) and industry and health care workers (21%) (21,49). In our study, JSS mean score was similar to a large cohort study in Finland (40). Occasional insomnia symptoms (any insomnia symptom 1–4 nights per month) were reported by 46% and insomnia disorder (any insomnia symptom ≥5 nights per week) by 10% of participants; the readings are very similar to those of a previous Finnish study (45% and 10%, respectively) (50). ECEC professionals spent 58% of their waking time sedentary and 42% in physical activity of which almost 90% was in light intensity. Proportions are very similar to earlier studies reporting accelerometer-derived physical activity levels in adult populations (51,52).

Our results on relation between recovery from work and insomnia are in line with previous studies on this topic, where the population was slightly younger and, unlike our study, included both women and men (16,17). Recovery from work associated with several insomnia symptoms (waking up several times per night, difficulties staying asleep, and non-restorative sleep) reflecting diverse deficiencies with sleep health that might challenge recovery. Sleep disturbances have associated with sickness absence and even one night of sleep deprivation increases the need for recovery for the following days (16,53). Hence, it might be valuable to explore ways to support unwinding from work already at an early stage of perceived need for recovery, in order to prevent insomnia and further to promote recovery. However, only few studies have explored the association between recovery from work and insomnia. More studies focusing on sleep health and recuperation from work in promoting recovery are warranted. Also, longitudinal studies are needed to examine cause-and-effect relationships in more detail.

We found that, in addition to insomnia, physical activity during working hours was associated with the need to recover from work. Results also suggested that if OPA was high the relationship between NFR and JSS was low to moderate, and if OPA was low the relationship was low or non-existing. These results support our hypothesis that high OPA could deteriorate recovery. It indicates that even low to moderate ECEC centre workload may be relevant in this context. Nevertheless, the findings

For peer review only - http://bmjopen.bmj.com/site/about/guidelines.xhtml

need to be interpreted cautiously. The result may be due to the poor physical condition of the participants as physically demanding work and sleep difficulties have been found to associate with poorer physical function (54,55). We did not measure participants' physical function; therefore, it remains unclear whether even a light workload, especially if long-lasting, may be too wearing and increase the need for recovery from work when one's physical condition is weak. Also, we noticed that low OPA was related to a higher level of education of participants. Employees in ECEC centres, who engage in higher levels of physical activity during work hours often hold roles that involve persistent interaction with children throughout the day, in comparison to their more educated counterparts. This job characteristic with active engagement and responsibility for children may intensify the perceived need for recuperation after working hours. OPA did not relate to other characteristics of participants. Also, we investigated only women, and they all worked in the same occupational setting (i.e. an ECEC centre). The research should be repeated with men, as well as with different professions, including those with more physically demanding work tasks. In addition, there is a potential limitation in representativeness of the study sample as this study is a secondary analysis from the DagisWork study, a randomized controlled intervention trial and we used a baseline data for this analysis.

The results suggest that LTPA is not relevant to the relationship between insomnia and recovery from work. In addition, the career history in ECEC center was related to LTPA, although age was not. High LTPA has been linked to better sleep (27,56) while the appropriate duration and intensity of physical activity are still debated (27). It has also been suggested that a high LTPA may protect from insomnia even when activity during the working day is high (33). However, this was not confirmed by our results and our hypothesis of recovery enhancing role of LTPA was not affirmed by the data. In some studies, high leisure-time physical activity has been linked to a lower need for recovery (20,57). Theoretically, we can assume that LTPA improves physical fitness, which increases stress tolerance and thereby reduces the need for recovery from work. On the other hand, LTPA may facilitate relaxation, promote psychological detachment from work and enhance experience of having autonomy and control over leisure-time choices and activities (3). However, our results did not support these assumptions. First, this may be due to the fact that accelerometer-derived LTPA inadequately captured participants' leisure-time activities performed in stationary positions, such as strength training. Second, an accelerometer may not be able to capture activities unwinding from work, such as relaxation exercises or Pilates-type training, for example. Third, the intensity of LTPA among ECEC workers may be too low to improve physical fitness and thereby increase load tolerance at work. They spent 58% of their waking time sedentary, and only 5% in

For peer review only - http://bmjopen.bmj.com/site/about/guidelines.xhtml

moderate or vigorous physical activity, similar proportions as a recent large cohort study reported (58). Also, the only characteristic related to LTPA was the career history in ECEC center, which we cannot explain, especially when age wasn't one of those factors. Further, what forms of exercise would best enhance recovery from work remains unknown. In the future, research should consider different domains of LTPA suitable at an individual level to enhance unwinding from work. For that, diary or similar methods would be needed parallel to technological measures. Recovery is a complex phenomenon that encompasses several facets of an individual's life, including their lifestyle, work, and various aspects related to the life circumstances. Significance of physical activity in this context remains an intriguing subject of study, exploring the type, amount and intensity of physical activity that supports recovery for individuals.

Conclusions

In conclusion, the results of this study suggest that both insomnia and physical activity during a working day are associated with recovery from work in ECEC professionals. Insomnia and recovery from work seem to relate especially if physical activity is high during a working day. This implies that ECEC professionals, especially those involved in high physical activity at work, should seek methods to improve their recovery, by incorporating activities that promote recuperation both during their workday and in their leisure time. Further research, particularly longitudinal studies, are warranted to explore the significance of both occupational physical activity and leisure-time physical activity separately as part of the individual and multifaceted entity of recovery.

References

1. Stynen D, Jansen NWH, Slangen JJM, de Grip A, Kant IJ. Need for recovery and different types of early labour force exit: a prospective cohort study among older workers. Int Arch Occup Environ Health. 2019 Jul;92(5):683–97.

2. van Hooff ML, Geurts SA, Taris TW, Kompier MA, Dikkers JS, Houtman IL, et al. Disentangling the causal relationships between work-home interference and employee health. Scand J Work Environ Health. 2005;31(1):15–29.

3. Sonnentag S, Cheng BH, Parker SL. Recovery from Work: Advancing the Field Toward the Future. Annu Rev Organ Psychol Organ Behav. 2022 Jan 21;9(1):33–60.

4. Stevens ML, Crowley P, Rasmussen CL, Hallman DM, Mortensen OS, Nygård CH, et al. Accelerometer-Measured Physical Activity at Work and Need for Recovery: A Compositional Analysis of Cross-sectional Data. Ann Work Expo Health. 2020 Feb 20;64(2):138–51.

For peer review only - http://bmjopen.bmj.com/site/about/guidelines.xhtml
For peer review only

5. Sonnentag S, Zijlstra FRH. Job characteristics and off-job activities as predictors of need for recovery, well-being, and fatigue. J Appl Psychol. 2006 Mar;91(2):330–50.

6. van Veldhoven M, Broersen S. Measurement quality and validity of the “need for recovery scale.” Occup Environ Med. 2003 Jun 1;60(>90001):3i–9.

7. Demerouti E, Bakker AB, Geurts SAE, Taris TW. Daily recovery from work-related effort during non-work time. In: Sonnentag S, Perrewé PL, Ganster DC, editors. Research in Occupational Stress and Well-being. Emerald Group Publishing Limited; 2009. p. 85–123.

8. Garrick A, Mak AS, Cathcart S, Winwood PC, Bakker AB, Lushington K. Non-Work Time Activities Predicting Teachers’ Work-Related Fatigue and Engagement: An Effort-Recovery Approach. Aust Psychol. 2018 Jun 1;53(3):243–52.

9. Xie L, Kang H, Xu Q, Chen MJ, Liao Y, Thiyagarajan M, et al. Sleep Drives Metabolite Clearance from the Adult Brain. Science. 2013 Oct 18;342(6156):373–7.

10. Cabeza de Baca T, Chayama KL, Redline S, Slopen N, Matsushita F, Prather AA, et al. Sleep debt: the impact of weekday sleep deprivation on cardiovascular health in older women. Sleep. 2019 Jul 30;zsz149.

11. Cappuccio FP, Cooper D, D’Elia L, Strazzullo P, Miller MA. Sleep duration predicts cardiovascular outcomes: a systematic review and meta-analysis of prospective studies. Eur Heart J. 2011 Jun;32(12):1484–92.

12. Hertenstein E, Feige B, Gmeiner T, Kienzler C, Spiegelhalder K, Johann A, et al. Insomnia as a predictor of mental disorders: A systematic review and meta-analysis. Sleep Med Rev. 2019 Feb;43:96–105.

13. Jike M, Itani O, Watanabe N, Buysse DJ, Kaneita Y. Long sleep duration and health outcomes: A systematic review, meta-analysis and meta-regression. Sleep Med Rev. 2018 Jun;39:25–36.

14. Wu TT, Zou YL, Xu KD, Jiang XR, Zhou MM, Zhang SB, et al. Insomnia and multiple health outcomes: umbrella review of meta-analyses of prospective cohort studies. Public Health. 2023 Feb;215:66–74.

15. Bergman E, Löyttyniemi E, Myllyntausta S, Rautava P, Korhonen PE. Factors associated with quality of life and work ability among Finnish municipal employees: a cross-sectional study. BMJ Open. 2020 Sep;10(9):e035544.

16. Lallukka T, Kaikkonen R, Härkänen T, Kronholm E, Partonen T, Rahkonen O, et al. Sleep and Sickness Absence: A Nationally Representative Register-Based Follow-Up Study. Sleep. 2014 Sep 1;37(9):1413–25.

17. Rook JW, Zijlstra FRH. The contribution of various types of activities to recovery. Eur J Work Organ Psychol. 2006 Jun;15(2):218–40.

18. Silva-Costa A, Griep RH, Fischer FM, Rotenberg L. Need for recovery from work and sleep-related complaints among nursing professionals. Work. 2012;41:3726–31.

19. Karihtala T, Valtonen AM, Kautiainen H, Hopsu L, Halonen J, Heinonen A, et al. Relationship between occupational and leisure-time physical activity and the need for recovery after work. Arch Public Health. 2023 Feb 10;81(1):17.

For peer review only - http://bmjopen.bmj.com/site/about/guidelines.xhtml

20. Coffeng JK, van Sluijs EM, Hendriksen IJM, van Mechelen W, Boot CRL. Physical Activity and Relaxation During and After Work are Independently Associated With the Need for Recovery. J Phys Act Health. 2015 Jan;12(1):109–15.

21. Gommans FG, Jansen NWH, Mackey MG, Stynen D, de Grip A, Kant Ij. The Impact of Physical Work Demands on Need for Recovery, Employment Status, Retirement Intentions, and Ability to Extend Working Careers: A Longitudinal Study Among Older Workers. J Occup Environ Med. 2016 Apr;58(4):e140–51.

22. Korpela K, Kinnunen U. How Is Leisure Time Interacting with Nature Related to the Need for Recovery from Work Demands? Testing Multiple Mediators. Leis Sci. 2010 Dec 30;33(1):1–14.

23. van Hooff MLM, Benthem de Grave RM, Geurts SAE. No pain, no gain? Recovery and strenuousness of physical activity. J Occup Health Psychol. 2019 Oct;24(5):499–511.

24. Steene-Johannessen J, Anderssen SA, Van Der Ploeg HP, Hendriksen IJM, Donnelly AE, Brage S, et al. Are Self-report Measures Able to Define Individuals as Physically Active or Inactive? Med Sci Sports Exerc. 2016 Feb;48(2):235–44.

25. Van Poel E, Ketels M, Clays E. The association between occupational physical activity, psychosocial factors and perceived work ability among nurses. J Nurs Manag. 2020 Oct;28(7):1696–703.

26. Fank F, Pereira F da S, dos Santos L, de Mello MT, Mazo GZ. Effects of Exercise on Sleep in Older Adults: An Overview of Systematic Reviews and Meta-Analyses. J Aging Phys Act. 2022;1–17.

27. Wang F, Boros S. The effect of physical activity on sleep quality: a systematic review. Eur J Physiother. 2021 Jan 2;23(1):11–8.

28. Wendt A, Flores TR, Silva ICM, Wehrmeister FC. Association of physical activity with sleep health: A systematic review. Rev Bras Atividade Física Saúde. 2018;23:1–26.

29. Atoui S, Chevance G, Romain AJ, Kingsbury C, Lachance JP, Bernard P. Daily associations between sleep and physical activity: A systematic review and meta-analysis. Sleep Med Rev. 2021 Jun 1;57:101426.

30. Memon AR, Gupta CC, Crowther ME, Ferguson SA, Tuckwell GA, Vincent GE. Sleep and physical activity in university students: A systematic review and meta-analysis. Sleep Med Rev. 2021 Aug;58:101482.

31. Beak M, Choi WJ, Lee W, Ham S. Associations of Abnormal Sleep Duration with Occupational and Leisure-time Physical Activity in the Working Population: A Nation-wide Population-based Study. Saf Health Work. 2021 Sep 1;12(3):311–6.

32. Lim M, Jeong KS, Oh SS, Koh SB, Chang SJ, Ahn YS. Effects of Occupational and Leisure-Time Physical Activities on Insomnia in Korean Firefighters. Int J Environ Res Public Health Electron Resour. 2020;17(15).

33. Skarpsno ES, Mork PJ, Nilsen TIL, Jørgensen MB, Holtermann A. Objectively measured occupational and leisure-time physical activity: cross-sectional associations with sleep problems. Scand J Work Environ Health. 2018 Nov 2;202–211.

For peer review only

For peer review only - http://bmjopen.bmj.com/site/about/guidelines.xhtml

34. Skarpsno ES, Mork PJ, Nilsen TIL, Jørgensen MB, Holtermann A. The joint association of musculoskeletal pain and domains of physical activity with sleep problems: cross-sectional data from the DPhacto study, Denmark. Int Arch Occup Environ Health. 2019 May;92(4):491–9.

35. Wennman H, Kronholm E, Partonen T, Tolvanen A, Peltonen M, Vasankari T, et al. Physical activity and sleep profiles in Finnish men and women. BMC Public Health. 2014 Dec;14(1):82.

36. de Croon EM, Sluiter JK, Frings-Dresen MHW. Psychometric properties of the Need for Recovery after work scale: test-retest reliability and sensitivity to detect change. Occup Environ Med. 2006 Mar 1;63(3):202–6.

37. Jenkins CD, Stanton BA, Niemcryk SJ, Rose RM. A scale for the estimation of sleep problems in clinical research. J Clin Epidemiol. 1988;41(4):313–21.

38. Lallukka T, Dregan A, Armstrong D. Comparison of a Sleep Item From the General Health Questionnaire-12 With the Jenkins Sleep Questionnaire as Measures of Sleep Disturbance. J Epidemiol. 2011;21(6):474–80.

39. Tibubos AN, Zenger M, Schmalbach B, Beutel ME, Brähler E. Measurement invariance, validation and normative data of the Jenkins Sleep Scale-4 (JSS-4) in the German general population across the life span. J Psychosom Res. 2020 Mar;130:109933.

40. Juhola J, Arokoski JPA, Ervasti J, Kivimäki M, Vahtera J, Myllyntausta S, et al. Internal consistency and factor structure of Jenkins Sleep Scale: cross-sectional cohort study among 80 000 adults. BMJ Open. 2021 Jan 1;11(1):e043276.

41. Choi L, Liu Z, Matthews CE, Buchowski MS. Validation of Accelerometer Wear and Nonwear Time Classification Algorithm. Med Sci Sports Exerc. 2011 Feb;43(2):357–64.

42. Freedson PS, Melanson E, Sirard J. Calibration of the Computer Science and Applications, Inc. accelerometer. Med Sci Sports Exerc. 1998 May;30(5):777.

43. Ilmarinen J. Work ability—a comprehensive concept for occupational health research and prevention. Scand J Work Environ Health. 2009 Jan;35(1):1–5.

44. Goldberg DP, Gater R, Sartorius N, Ustun TB, Piccinelli M, Gureje O, et al. The validity of two versions of the GHQ in the WHO study of mental illness in general health care. Psychol Med. 1997 Jan;27(1):191–7.

45. Ware JE, Sherbourne CD. Erratum to The MOS 36-item short form health survey (SF-36): I. Conceptual framework. PharmacoEconomics. 1992 Aug;2(2):98–98.

46. Cohen S, Kamarck T, Mermelstein R. A Global Measure of Perceived Stress. J Health Soc Behav. 1983 Dec;24(4):385.

47. Graham B, Cottey L, Smith JE, Mills M, Latour JM. Measuring 'Need for Recovery' as an indicator of staff well-being in the emergency department: a survey study. Emerg Med J. 2020 Sep;37(9):555–61.

48. Hemiö K, Lindström J, Peltonen M, Härmä M, Viitasalo K, Puttonen S. High need for recovery from work and sleep problems are associated with workers' unhealthy dietary habits. Public Health Nutr. 2021 Jun;24(8):2185–94.

For peer review only

For peer review only - http://bmjopen.bmj.com/site/about/guidelines.xhtml

49. Qi X, Liu Y, Zhang J, Ji S, Sluiter JK, Zhou R, et al. Relationship between work strain, need for recovery after work and cumulative cortisol among kindergarten teachers. Int Arch Occup Environ Health. 2015 Nov;88(8):1053–9.

50. Kronholm E, Partonen T, Härmä M, Hublin C, Lallukka T, Peltonen M, et al. Prevalence of insomnia-related symptoms continues to increase in the Finnish working-age population. J Sleep Res. 2016;25(4):454–7.

51. Ekblom-Bak E, Börjesson M, Bergman F, Bergström G, Dahlin-Almevall A, Drake I, et al. Accelerometer derived physical activity patterns in 27.890 middle-aged adults: The SCAPIS cohort study. Scand J Med Sci Sports. 2022 May;32(5):866–80.

52. Husu P, Suni J, Vähä-Ypyä H, Sievänen H, Tokola K, Valkeinen H, et al. Objectively measured sedentary behavior and physical activity in a sample of Finnish adults: a cross-sectional study. BMC Public Health. 2016 Dec;16(1):920.

53. Laharnar N, Fatek J, Zemann M, Glos M, Lederer K, Suvorov AV, et al. A sleep intervention study comparing effects of sleep restriction and fragmentation on sleep and vigilance and the need for recovery. Physiol Behav. 2020 Mar;215:112794.

54. Aikomus L, Lehtinen-Jacks S, Nordquist H, Pietiläinen O, Mänty M, Kouvonen A, et al. Työn fyysisen kuormittavuuden yhteys fyysiseen toimintakykyyn alle 40-vuotiailla kunta-alan työntekijöillä. Sos Aikakauslehti. 2021 Dec 16;58(4).

55. Miranda H, Viikari-Juntura E, Punnett L, Riihimäki H. Occupational loading, health behavior and sleep disturbance as predictors of low-back pain. Scand J Work Environ Health. 2008 Dec;34(6):411–9.

56. Bláfoss R, Sundstrup E, Jakobsen MD, Bay H, Garde AH, Andersen LL. Are Insomnia Type Sleep Problems Associated With a Less Physically Active Lifestyle? A Cross-Sectional Study Among 7,700 Adults From the General Working Population. Front Public Health. 2019 May 14;7:117.

57. Wilson I, McDermott H, Munir F. The role of working hours, work environment and physical leisure activity on the need for recovery following a day's work among UK white-water raft guides: A within-subjects multilevel approach. Psychol Sport Exerc. 2016 Mar;23:123–31.

58. Guthold R, Stevens GA, Riley LM, Bull FC. Worldwide trends in insufficient physical activity from 2001 to 2016: a pooled analysis of 358 population-based surveys with 1·9 million participants. Lancet Glob Health. 2018 Oct 1;6(10):e1077–86.

Legends for figures:

Figure 1. Study profile

Figure 2. Daily minutes of physical activity categorized into four groups based on median splits of leisure time physical activity (LTPA min/day) and occupational physical activity (OPA min/day): A) high LTPA + low OPA, B) high LTPA + high OPA, C) low LTPA + low OPA and D) low LTPA +

For peer review only

high OPA. Medians for LTPA (246.2 min/day) and for OPA (231.0 min/day) are illustrated as dashed lines.

Figure 3. Age-adjusted correlations with 95% CIs between Need for Recovery and Jenkins Sleep Scale for the physical activity groups based on low or high leisure-time physical activity (LTPA, min/day) and occupational physical activity (OPA, min/day): high LTPA + low OPA, high LTPA + high OPA, low LTPA + low OPA or low LTPA + high OPA.

Figure 4. Multivariate relationships between Need for Recovery (NFR) and occupational physical activity (OPA) level, leisure-time physical activity (LTPA) level, age, and Jenkins Sleep Scale (JSS) sum score. Cohen's standards for beta values above 0.10, 0.30, and 0.50 represent small, moderate, and large effect sizes, respectively.

For peer review only

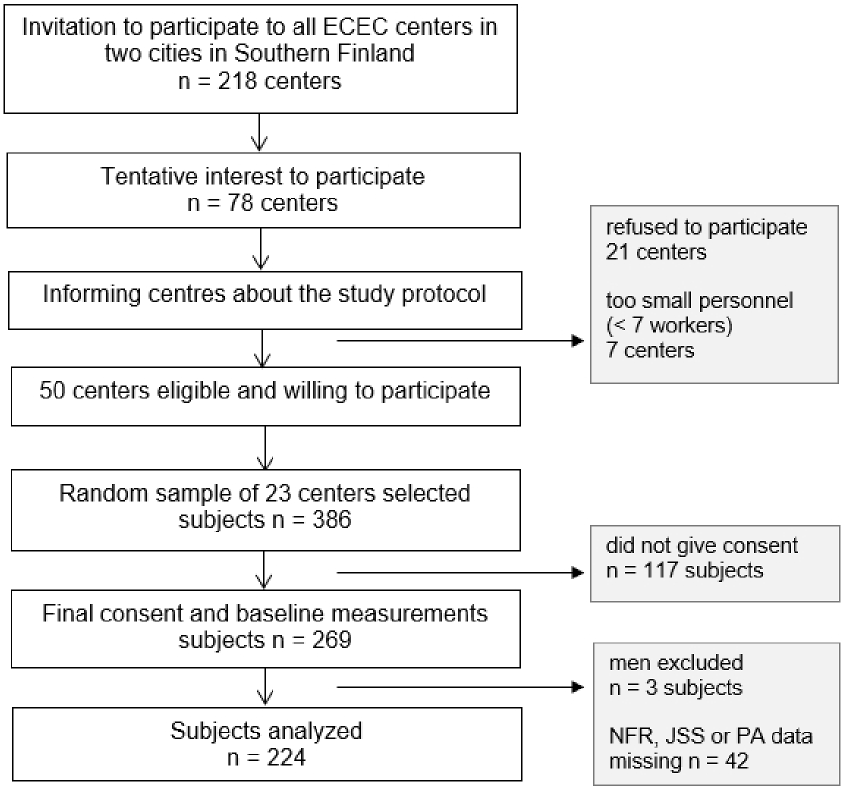

Figure 1. Study profile

303x270mm (57 x 57 DPI)

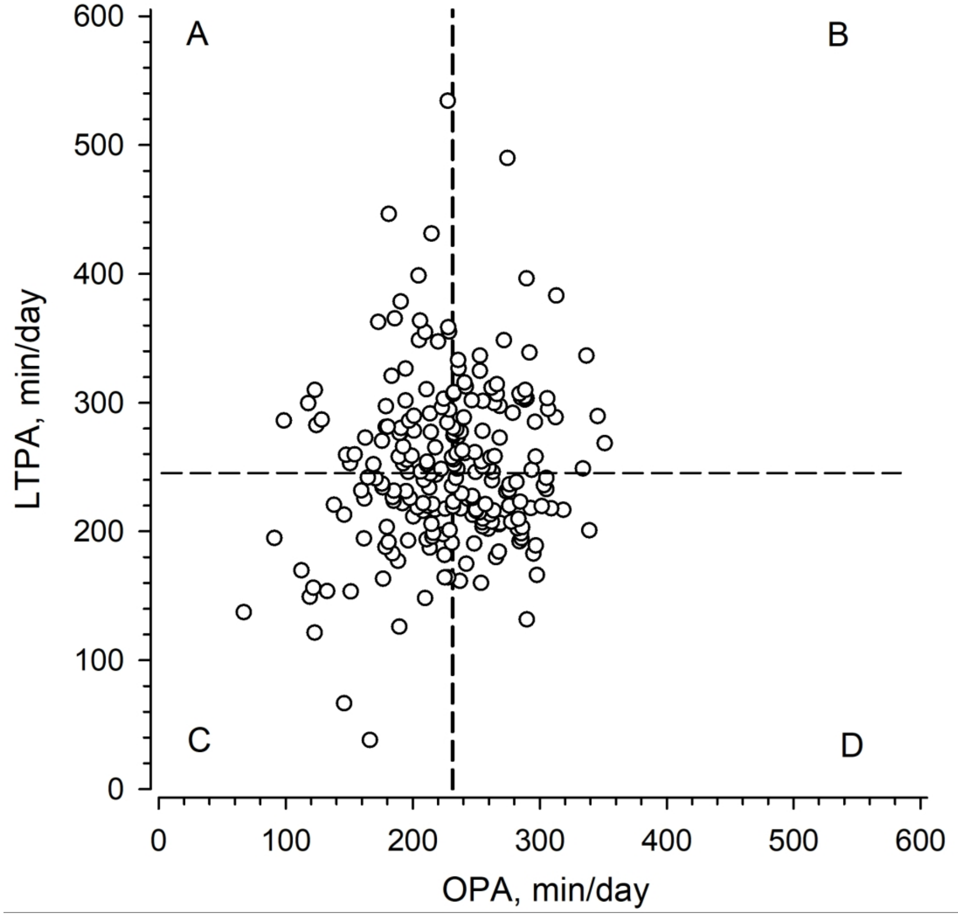

Figure 2. Daily minutes of physical activity categorized into four groups based on median splits of leisure time physical activity (LTPA min/day) and occupational physical activity (OPA min/day): A) high LTPA + low OPA, B) high LTPA + high OPA, C) low LTPA + low OPA and D) low LTPA + high OPA. Medians for LTPA (246.2 min/day) and for OPA (231.0 min/day) are illustrated as dashed lines.

226x208mm (236 x 236 DPI)

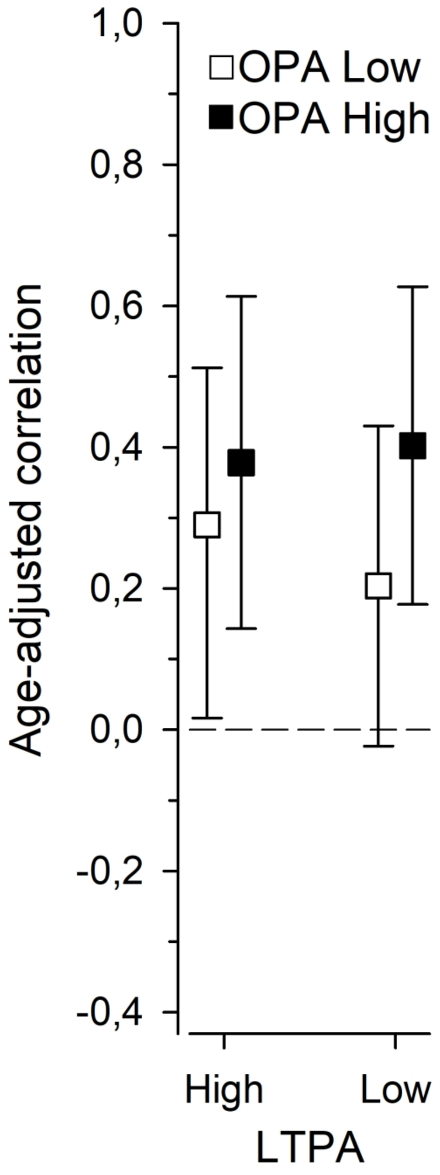

Figure 3. Age-adjusted correlations with 95% CIs between Need for Recovery and Jenkins Sleep Scale for the physical activity groups based on low or high leisure-time physical activity (LTPA, min/day) and occupational physical activity (OPA, min/day): high LTPA + low OPA, high LTPA + high OPA, low LTPA + low OPA or low LTPA + high OPA.

105x271mm (236 x 236 DPI)

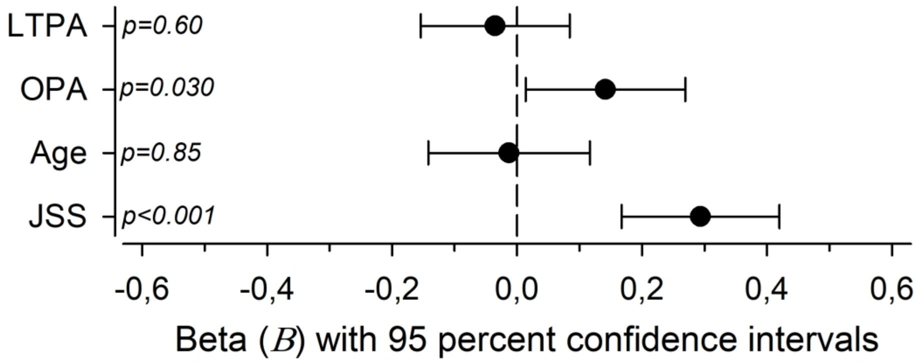

Figure 4. Multivariate relationships between Need for Recovery (NFR) and occupational physical activity (OPA) level, leisure-time physical activity (LTPA) level, age, and Jenkins Sleep Scale (JSS) sum score. Cohen's standards for beta values above 0.10, 0.30, and 0.50 represent small, moderate, and large effect sizes, respectively.

278x133mm (236 x 236 DPI)

STROBE checklist

Section/Topic	Item #	Recommendation	Reported on page #
**Title and abstract**	1	(*a*) Indicate the study's design with a commonly used term in the title or the abstract	1
		(*b*) Provide in the abstract an informative and balanced summary of what was done and what was found	1
**Introduction**			
Background/rationale	2	Explain the scientific background and rationale for the investigation being reported	3 and 4
Objectives	3	State specific objectives, including any prespecified hypotheses	4
**Methods**			
Study design	4	Present key elements of study design early in the paper	4
Setting	5	Describe the setting, locations, and relevant dates, including periods of recruitment, exposure, follow-up, and data collection	4
Participants	6	(*a*) Give the eligibility criteria, and the sources and methods of selection of participants	4
Variables	7	Clearly define all outcomes, exposures, predictors, potential confounders, and effect modifiers. Give diagnostic criteria, if applicable	5 and 6
Data sources/ measurement	8*	For each variable of interest, give sources of data and details of methods of assessment (measurement). Describe comparability of assessment methods if there is more than one group	5,6 and 7
Bias	9	Describe any efforts to address potential sources of bias	4
Study size	10	Explain how the study size was arrived at	4
Quantitative variables	11	Explain how quantitative variables were handled in the analyses. If applicable, describe which groupings were chosen and why	4,5 and 6
Statistical methods	12	(*a*) Describe all statistical methods, including those used to control for confounding	7
		(*b*) Describe any methods used to examine subgroups and interactions	7
		(*c*) Explain how missing data were addressed	4
		(*d*) If applicable, describe analytical methods taking account of sampling strategy	4
		(*e*) Describe any sensitivity analyses	NA

For peer review only

**Results**			
Participants	13*	(a) Report numbers of individuals at each stage of study—eg numbers potentially eligible, examined for eligibility, confirmed eligible, included in the study, completing follow-up, and analysed	4 and Figure 1
		(b) Give reasons for non-participation at each stage	4
		(c) Consider use of a flow diagram	Figure 1
Descriptive data	14	(a) Give characteristics of study participants (eg demographic, clinical, social) and information on exposures and potential confounders	9
		(b) Indicate number of participants with missing data for each variable of interest	4
Outcome data	15	Report numbers of outcome events or summary measures	NA
Main results	16	(*a*) Give unadjusted estimates and, if applicable, confounder-adjusted estimates and their precision (eg, 95% confidence interval). Make clear which confounders were adjusted for and why they were included	8 and 10
		(*b*) Report category boundaries when continuous variables were categorized	8
		(*c*) If relevant, consider translating estimates of relative risk into absolute risk for a meaningful time period	NA
Other analyses	17	Report other analyses done—eg analyses of subgroups and interactions, and sensitivity analyses	NA
**Discussion**			
Key results	18	Summarise key results with reference to study objectives	10 and 11
Limitations	19	Discuss limitations of the study, taking into account sources of potential bias or imprecision. Discuss both direction and magnitude of any potential bias	11 and 12
Interpretation	20	Give a cautious overall interpretation of results considering objectives, limitations, multiplicity of analyses, results from similar studies, and other relevant evidence	11 and 12
Generalisability	21	Discuss the generalisability (external validity) of the study results	11 and 12
**Other information**			
Funding	22	Give the source of funding and the role of the funders for the present study and, if applicable, for the original study on which the present article is based	2

For peer review only
